# Single-cell/spatial integration reveals an MES2-like glioblastoma program orchestrated by immune communication and regulatory networks

**DOI:** 10.3389/fimmu.2025.1699134

**Published:** 2025-10-29

**Authors:** Chonghui Zhang, Lu Tan, Kaijian Zheng, Yifan Xu, Junshan Wan, Jinpeng Wu, Chao Wang, Pin Guo, Yugong Feng

**Affiliations:** ^1^ Department of Neurosurgery, The Affiliated Hospital of Qingdao University, Qingdao, China; ^2^ Institute of Neuroscience & Jiangsu Key Laboratory of Neuropsychiatric Diseases, Soochow University, Suzhou, Jiangsu, China; ^3^ Qingdao Central Hospital, University of Health and Rehabilitation Sciences, Qingdao, China

**Keywords:** MES2-like glioblastoma, cell type-aware graph neural network, TAM-MG-MES2 communication, spatial transcriptome analysis, arrestin domain containing 3

## Abstract

**Background:**

Glioblastoma (GBM) exhibits marked plasticity and intense microenvironmental crosstalk. We aimed to delineate mesenchymal programs with spatial resolution, clinical relevance, and mechanistic anchors.

**Methods:**

We integrated single-cell RNA-seq, bulk transcriptomes, and Visium spatial data. After rigorous QC and Harmony integration, we annotated 12 cell states using canonical markers, decoupler-based ORA, and AUCell. Tumor boundaries were defined by inferCNV/CopyKAT; developmental potential by CytoTRACE2 and PHATE. Post-translational modification (PTM) axes were scored from curated gene sets. A cell type-aware GNN linked bulk expression to a patient-similarity graph for survival modeling and gene-level hazard attribution. Network convergence combined bulk WGCNA (TCGA/CGGA), single-cell hdWGCNA, BayesPrism deconvolution, and external GEO validation. Ligand–receptor (LR) signaling was inferred with LIANA+, embedded in a signed causal network, and mapped spatially. ARRDC3 expression was assessed in GBM tissues; U251 gain- and loss-of-function assays evaluated proliferation and migration.

**Results:**

We resolved major GBM states, including two mesenchymal programs (MES1-like, MES2-like). CNV-high regions marked malignant cores, and CytoTRACE2 identified high-potency niches within MES2-like and Proliferation states along non-linear trajectories. PTM landscapes segregated by state; S-nitrosylation, glycosylation, and lactylation were enriched in mesenchymal programs. A GNN risk score stratified overall survival in TCGA (n=157) and generalized to CGGA-325 (n=85) and CGGA-693 (n=140). MES2-like abundance remained an independent adverse predictor (HR = 2.31; 95% CI, 1.04–5.10). MES2-high tumors upregulated EMT, TNFα/NF-κB, JAK/STAT, hypoxia, angiogenesis, and glycolysis; S-nitrosylation associated with increased hazard. Cross-modal convergence defined a conservative MES2 core enriched for ECM remodeling, collagen modification, focal adhesion, and TGF-β regulation. LR analysis prioritized a TAM-to-MES2 axis (e.g., GRN–TNFRSF1A, ADAM9/10/17–ITGB1, TGFB1–ITGB1/EGFR) converging on a CEBPD-centered module. Spatial mapping localized MES2 hotspots within CNV-defined territories and revealed a TNFRSF1A–CEBPD–ARRDC3 focus at an infiltrative rim. ARRDC3 was upregulated in GBM tissues; in U251 cells, knockdown promoted and overexpression suppressed proliferation and migration, indicating context-dependent roles.

**Conclusions:**

MES2-like GBM is an ECM-driven, stress-adapted state with strong prognostic impact. We nominate CEBPD and TNFRSF1A/ITGB1 as actionable nodes and identify ARRDC3 as a spatially restricted effector with context-dependent tumor-modulatory functions warranting therapeutic exploration.

## 1 Introduction

Glioblastoma (GBM) is an exceptionally aggressive and heterogeneous brain tumor that poses a formidable challenge in neuro-oncology, with limited therapeutic options and poor prognosis (1). Although significant progress has been made in understanding glioma biology, the complex molecular networks driving GBM invasiveness remain insufficiently characterized (2). A defining feature of GBM pathophysiology is its intricate tumor microenvironment (TME), which not only supports tumor growth but also contributes to therapeutic resistance and recurrence (3, 4).

Recent studies have emphasized the dynamic interactions between tumor cells and various cellular components within the microenvironment, including immune cells, endothelial cells, and stromal elements, that collectively regulate tumor progression (5). Among the molecular subtypes of GBM, the mesenchymal (MES) subtype has emerged as a key determinant of malignancy, characterized by pronounced plasticity, enhanced invasiveness, and resistance to conventional therapies (6). Importantly, GBM cells rarely exist in fixed cellular states; instead, they exhibit remarkable plasticity, transitioning between transcriptional programs in response to microenvironmental cues and therapeutic pressures. Within this mesenchymal category, recent high-dimensional analyses have further dissected the phenotype into distinct MES1- and MES2-like states with unique molecular signatures (7). Notably, the MES2-like program is marked by adaptations to hypoxia and inflammatory signaling, representing a dynamic and invasive tumor subpopulation intimately associated with disease progression (8). However, the regulatory circuits and microenvironmental interactions that generate and sustain the MES2-like state remain poorly understood.

This knowledge gap is partly attributable to inherent limitations of bulk profiling methods, which obscure the subtle heterogeneity and spatial organization within tumors. The advent of single-cell RNA sequencing (scRNA-seq) has revolutionized neuroscience and oncology by providing cell-resolved insights into the tumor ecosystem, enabling precise delineation of tumor and stromal phenotypes (9). Complementing this, spatial transcriptomics preserves tissue architecture while interrogating gene expression, allowing analysis of cellular neighborhoods, spatially constrained signaling, and niche-specific regulatory events (10). Integrating single-cell and spatial transcriptomics thus holds promise for elucidating the complex crosstalk between tumor cell states and their microenvironment, particularly the interactions driving mesenchymal transition.

Emerging evidence implicates a MES2-like mesenchymal program as a driver of invasion, stress adaptation, and therapy resistance in GBM, yet its regulatory circuitry, spatial niches, and immunologic dependencies remain unresolved. We therefore set out to delineate the core MES2-like network, map its localization within tumor ecosystems, and identify upstream cues and transcriptional effectors that could be leveraged to rewire the mesenchymal state.

## 2 Methods

### 2.1 Data sources

**Figure 1:**
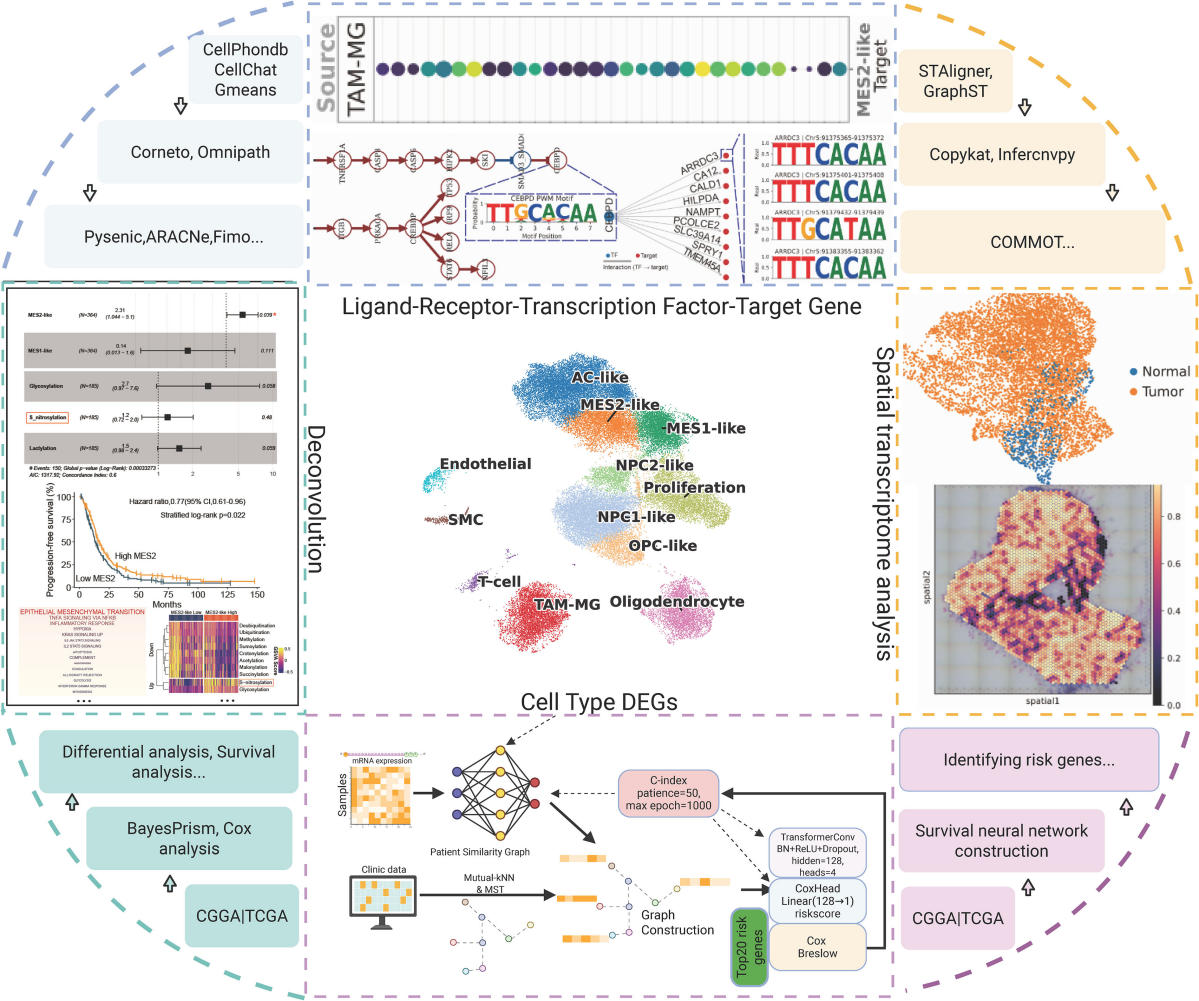
Research design framework.

Single-cell transcriptomic data were downloaded from GEO under accession numbers GSE103224 (8 GBM samples), GSE138794 (20 GBM samples), and GSE139448 (6 GBM samples). Spatial transcriptomic data were obtained from GSE194329, of which four samples were analyzed in detail (GBM2: IDH-wt recurrent tumor; GBM3: IDH-wt primary tumor; GBM5_1: IDH-wt primary tumor; GBM5_2: IDH-wt peri-tumoral tissue). Bulk RNA-seq data were obtained from TCGA (157 GBM samples) and from the Chinese Glioma Genome Atlas (CGGA mRNA325, 85 GBM; CGGA mRNA693, 140 GBM). Additional microarray cohorts were included from GSE4290 (77 GBM, 23 normal) and GSE68848 (228 GBM, 28 normal). The overall research design process is shown in **Figure 1**.

### 2.2 Single cell processing

Raw FASTQ files were processed with 10x Genomics Cell Ranger (11), alignment to Human reference GRCh38 (2024-A). Protein-coding genes were annotated using the GENCODE v47 reference genome. To ensure data quality, we excluded samples with insufficient coverage (<1,000 detected cells) or low complexity (<500 detected genes). After quality control, 21 out of the initial 34 GBM single-cell samples were retained for downstream analysis. Single-cell RNA-seq data were processed with Scanpy (12). Raw counts were normalized to a fixed library size and log-transformed, followed by selection of 2,000 highly variable genes across samples. Gene expression values were scaled, and dimensionality reduction was performed using principal component analysis(PCA). We clustered and evaluated the performance of various batch correction methods (Harmony, scanorama, combat) within the principal component representation (PCR) space. Based on a proximity graph, we used the leiden algorithm for multi-scale clustering at resolutions ranging from 0.3-0.9. Benchmarker was then used to systematically evaluate the results at each resolution, comprehensively examining biological fidelity and batch effect removal (13). Batch effects were corrected with Harmony integration, after which a neighborhood graph was constructed. Cells were clustered with the leiden algorithm (resolution 0.8). To focus on robust clusters, only clusters containing at least 300 cells were retained. Cell-type markers for GBM were obtained from previously published article (**Supplementary Table 1**) (14). Using decoupler (15), we applied over-representation analysis (ORA) and AUCell to score the activity of marker gene sets (≥3 markers per cell type). Gene sets for GO Biological Processes (BP) and post-translational modification (PTM) enzymes were obtained from MsigDB (https://www.gsea-msigdb.org/gsea/msigdb) and previously published article (**Supplementary Table 2**) (16). Enrichment scores were estimated per cell type, and differential activity was assessed with a moderated t-test, retaining pathways with adjusted *p* < 0.05.

### 2.3 Tumor cell recognition

Copy number variation (CNV) inference was performed using the infercnvpy (17). Cells from GBM-associated states were analyzed, while non-tumor cells (Such as endothelial cells and T cells) served as reference “normal” populations. CNV profiles were estimated using a 250-gene sliding window. For classification, CNV scores were calculated for all cells, and the mean score of reference cells plus 1.5 standard deviations was used as the threshold to identify cells with elevated CNV as putative tumor cells. Tumor cells were subjected to functional profiling. Pathway activities were estimated with PROGENy gene sets and enrichment of custom molecular modification signatures was assessed by ORA (16). Significant pathways and PTM score (adjusted p < 0.05) were identified per cell type.

### 2.4 Differentiation potential inference

Cellular differentiation potential was inferred using CytoTRACE2 (18). Default parameters were applied with a fixed random seed to ensure reproducibility. The resulting CytoTRACE2 scores were embedded using PHATE for 3D visualization (19). Cell-type identities and CytoTRACE2 scores were overlaid on the PHATE embeddings to illustrate lineage hierarchies and differentiation gradients across GBM cell populations.

### 2.5 scRNA deconvolution, survival and functional analysis

Bulk RNA-seq datasets from TCGA and CGGA cohorts were batch-corrected using ComBat-seq (20), and cell-type proportions were inferred with BayesPrism using tumor cell states (21). Associations between deconvolved tumor cell states and patient survival were evaluated by Cox proportional hazards regression, including both univariate and multivariate models, with survival differences assessed by Kaplan-Meier analysis. For functional characterization, we stratified tumors by MES2-like abundance (above vs. below median) and performed differential expression analysis using DESeq2 (22). Significantly dysregulated genes were subjected to pathway enrichment against MSigDB hallmark gene sets. In parallel, gene sets representing post-translational modifications were scored using gene set variation analysis (GSVA), and their associations with clinical outcomes were further assessed through Cox regression.

### 2.6 MES2-like module definition based on bulk level

Weighted gene co-expression network analysis (WGCNA) was applied to bulk RNA-seq profiles (TCGA and CGGA) to identify co-expression modules associated with BayesPrism-inferred cell fractions. Soft-thresholding powers were selected using scale-free topology criteria, and modules were defined by hierarchical clustering and dynamic tree cutting. Module eigengenes were correlated with cell-type fractions, and genes from modules associated with MES2-like states were extracted. MES2-like module genes, defined as differentially upregulated genes in the high-MES2 group, was used to compute activity scores for the tumor cell type.

### 2.7 Graph construction and transformer-based survival modeling

To identify cell type-specific marker genes, we performed differential expression analysis across cell types. Genes were ranked within each cell type against all others by the Wilcoxon rank-sum test (log2FC>1 and adjust *P* < 0.05). Clinical data were used to construct patient-similarity networks, followed by appropriate preprocessing (min–max scaling, one-hot encoding, or rank scaling). Similarity matrices were derived using multiple metrics, including Gower distance, local-scaling kernels, and multi-view fusion, and graphs were subsequently built by retaining each patient’s five nearest neighbors with mutual k-Nearest Neighbors (kNN) filtering; to guarantee full connectivity, a minimum spanning tree backbone was added. Gene expression matrices were log_2_-transformed and standardized using training set statistics to avoid information leakage, and clinical survival metadata were matched by patient identifiers. To incorporate biological priors, genes were projected onto cell type signatures-level representations through a masked projector with residual connections, which were then modeled using a Transformer-based graph neural network. Survival prediction was formulated with a Cox proportional hazards head, trained under Adam optimization with dropout regularization and early stopping, and the best-performing model state was selected based on validation concordance index.

### 2.8 MES2-like module definition based on scRNA level

Cell type-specific co-expression networks were constructed using high-dimensional WGCNA (hdWGCNA) (23), after removing mitochondrial and ribosomal genes. This framework aggregates cells into metacells, optimizes soft-thresholding powers, and delineates distinct transcriptional modules. Module eigengenes were correlated with cell type and sequencing traits, and hub genes were defined based on intramodular connectivity (kME). Functional enrichment of ranked intramodular genes was performed using fgsea (v1.28) against GO BP pathways. Protein-protein interaction (PPI) support was integrated from the STRINGdb (v12.0) human network, retaining only experimentally supported edges. We further combined topological overlap matrices (TOM) from hdWGCNA with STRING PPI adjacency to construct integrative co-expression-PPI networks. Hub genes were defined as those with both high co-expression connectivity and PPI degree centrality.

### 2.9 Integrative identification of MES2 hub genes

To validate the robustness of MES2-associated signals, we systematically integrated bulk and scRNA resources. Two independent GEO cohorts (GSE4290 and GSE68848) were curated, and stringent outlier detection ensured removal of aberrant samples prior to downstream analysis. Differential expression was first profiled with limma (24). To guard against false positives driven by case–control imbalance, we complemented this with RankCompV2 (24), which detects genes showing consistent reversals in relative expression orderings (REOs) between gene pairs: REOs stable in normal brain defined the background, while GBM-specific reversals were assessed by Fisher’s exact test. Genes significant by the REO test were considered differential expression by RankCompV2, and only differentially expressed genes (DEGs) corroborated by both approaches were retained for downstream analyses, integrating absolute expression shifts with rank-based regulatory changes. We then converged multiple layers of evidence to define putative MES2 hub genes: WGCNA module, hdWGCNA module, scRNA DEGs from MES2-like clusters, bulk MES2-specific signatures inferred by deconvolution, and upregulated genes across both GEO cohorts. Intersections across these modalities yielded a conservative set of recurrent genes, representing a high-confidence MES2-like core program.

### 2.10 Regulatory network inference and transcription factor analysis

We applied the SCENIC workflow to infer gene regulatory networks (GRN) and transcription factors (TFs) activity at single-cell resolution. Raw UMI matrices were preprocessed with Scanpy, followed by pySCENIC (25), which integrates three modules: (i) GRN inference using pyscenic grn with a comprehensive set of human TFs to identify co-expression modules; (ii) cis-regulatory motif enrichment against curated motif and cisTarget databases to refine TF-target interactions; and (iii) regulon activity quantification, generating cell-by-regulon AUC matrices. Cell type-specific regulatory programs were derived by testing differential regulon activity across annotated clusters using Wilcoxon rank-sum tests. TFs significantly enriched in MES2-like (adjusted *P* < 0.05, |log_2_FC| > 1) were designated as up- or down-regulated master regulators. This approach enabled the identification of TFs shaping MES2-like states and provided a network-level perspective linking transcriptomic programs to regulatory control.

### 2.11 Cell-cell communication analysis

To systematically characterize the signaling crosstalk shaping MES2-like GBM states, we applied the LIANA+ framework (26), which integrates multiple LR inference methods including SingleCellSignalR, Connectome, CellPhoneDB, NATMI, logFC-based scoring, CellChat, and a geometric mean consensus. Significance was assessed with 1,000 permutations, and method-specific p-values were aggregated into a consensus ranking via LIANA’s meta-aggregation procedure.

We focused on interactions where macrophage/microglia (TAM-MG) served as the sender and MES2-like cells as the receiver. Candidate interactions were filtered to retain only those consistently significant across CellChat, CellPhoneDB, and consensus geometric mean (*P* < 0.05), with positive LR log_2_FC values indicating upregulated signaling. To further prioritize biologically relevant signals, predicted ligands and receptors were intersected with independent gene signatures of upregulated genes from bulk GBM datasets (GEO), thereby highlighting TAM-MG-MES2-like signaling axes supported by both single-cell inference and orthogonal bulk validation.

### 2.12 Causal signaling inference with CORNETO/CARNIVAL

To connect extracellular receptor activity with downstream transcriptional regulators of the MES2-like state, we integrated LR interaction scores with TF regulon specificity profiles.LR scores derived from LIANA+ were used as upstream inputs, while MES2- like-specific hub TFs, prioritized by regulon specificity scores (RSS) from pySCENIC, were designated as downstream outputs. Both input and output scores were provided as quantitative constraints to the CORNETO implementation of CARNIVAL (Unifying multi-sample network inference from prior knowledge and omics data with CORNETO (27), which optimizes causal signaling flows over the SIGNOR prior knowledge network (http://signor.uniroma2.it/).

### 2.13 Inference of CEBPD-MES2 regulons

To reconstruct transcriptional regulatory relationships specific to MES2-like cells, we applied ARACNe-AP (100 bootstraps, *P* < 1×e^-8^) (28). For motif-level validation, we retrieved transcription start sites (TSS) and promoter coordinates (−1 kb to +100 bp) of candidate targets from Ensembl Biomart and extracted corresponding genomic sequences (hg38). Position weight matrices (PWMs) for CEBPD were obtained from JASPAR2024, converted into MEME format, and scanned across promoter sequences using FIMO (29) (MEME Suite, *P* < 1e−4). Significant motif hits were then intersected with promoter regions of CEBPD-inferred targets, generating a refined set of direct regulatory candidates. Motif matches were annotated with genomic coordinates, binding scores, and associated target genes, providing sequence-level support for CEBPD-MES2-like regulatory edges.

### 2.14 Graph-based integration and spatial domain characterization

Visium spatial transcriptomic data from four GBM specimens (GBM2, GBM3, GBM5_1, GBM5_2) were processed using Scanpy for standard quality control, excluding spots with <200 detected genes or >25% mitochondrial content. Spatial graphs were constructed for each section with a 50 µm radius cutoff, and highly variable genes were selected before downstream analysis. Batch alignment across tissue sections was performed using STAligner (30), which embeds each slice as a subgraph and iteratively aligns paired sections. Optimal clustering resolution was determined by maximizing the silhouette score across leiden partitions. To distinguish malignant from non-malignant spots, large-scale CNV was inferred using CopyKAT and inferCNV (17, 31). For within-sample clustering, we applied GraphST with mclust-based refinement, ensuring spatial coherence of identified domains (32). Pathway activities were then inferred with decoupler (PROGENy framework), and spatial density maps were generated for MES2-like hub genes.

### 2.15 Spatial LR inference

To explore spatially organized cell–cell communication, we applied LIANA+ in a spatial mode to each GBM section. Normalized and log-transformed expression matrices were used as input, and spatial neighbor graphs were constructed with a Gaussian kernel (bandwidth = 200 µm, cutoff = 0.1). LR interactions were inferred using the consensus resource, integrating multiple published databases. For each candidate interaction, we computed both global (Moran’s I) and local (cosine similarity) statistics to assess spatial autocorrelation and co-enrichment, with significance determined by 100 random permutations. From the global LR atlas, we focused on a curated panel of interactions implicated in glioblastoma biology. Significant pairs (Moran’s I p < 0.05) were visualized as spatial feature maps, displaying both interaction scores and permutation-based p-values across tissue domains.

### 2.16 Clinical sample collection

This research was approved by the Ethics Committee of the Affiliated Hospital of Qingdao University (Approval Number: QYFYWZLL30508), and all participants provided written informed consent. A total of 6 patients diagnosed with glioblastoma (GBM) were enrolled in this study, with the control group consisting of normal brain tissue adjacent to the tumor. All tissue samples were stored in liquid nitrogen to preserve their integrity for subsequent molecular analysis.

### 2.17 Quantitative real-time PCR

Total RNA was extracted using Trizol reagent, and complementary DNA (cDNA) was synthesized with the Advantage RT for PCR Kit (Shandong Sparkjade Biotechnology Co., Ltd., China). Quantitative real-time PCR (qRT-PCR) analysis was subsequently performed using the iQTM SYBR Green Supermix (Yeasen Biotech Co., Ltd., China). Relative gene expression levels were calculated using the 2^−ΔΔCT^ method.

### 2.18 Western blot

Cellular and tissue proteins were extracted using RIPA buffer. Protein concentrations were determined using a BCA protein assay kit (Meilun, China). Equal amounts of protein were separated by electrophoresis on 10% SDS-PAGE gels and subsequently transferred onto polyvinylidene fluoride (PVDF) membranes (Thermo Fisher Scientific). The membranes were first incubated with a protein-free blocking buffer for 10 minutes, followed by overnight incubation at 4°C with primary antibodies against Arrestin Domain Containing 3 (ARRDC3, ab64817, Abcam, UK) and GAPDH (60004-1, Proteintech, China). Afterward, the membranes were incubated with horseradish peroxidase (HRP)-conjugated secondary antibodies for 50 minutes at room temperature. Protein bands were detected using enhanced chemiluminescence (ECL) reagents and visualized with a chemiluminescent imaging system (Millipore, USA).

### 2.19 Immunohistochemistry

Tissue sections were incubated with anti-ARRDC3 antibody (Proteintech Europe, Manchester, UK) overnight at 4°C. Colorimetric reactions were carried out in accordance with the manufacturer’s instructions (Thermo Scientific, Freemont, CA, USA) following washing and application of secondary antibodies. Nuclei were counterstained with hematoxylin (Carl Roth, Karlsruhe, Germany). Sections were digitized using a NIKON Eclipse Ti full-slide scanner.

The staining intensity of ARRDC3 was categorized as “negative,” “weak,” “moderate,” or “strong,” and assigned a score of 0, 1, 2, or 3, respectively. The proportion of positive cells was divided into four categories: 1 (<10%), 2 (11–50%), 3 (51–80%), and 4 (>80%). The immunohistochemical score was calculated as the product of staining intensity and the proportion of positive cells, yielding a total score ranging from 0 to 12.

### 2.20 Cell culture and lentivirus transfection.

U251 cells were cultured in DMEM medium supplemented with 10% fetal bovine serum at 37°C under a 5% CO_2_ atmosphere. The design and synthesis of ARRDC3 knockdown and overexpression shRNAs were conducted by OBiO Technology (Shanghai, China); detailed sequence information is provided in the **Supplementary Table 3**. The transfection procedure was carried out in accordance with the manufacturer’s instructions, and subsequent experimental assays were performed following transfection.

### 2.21 Cell counting kit−8 assay

Cells transfected with lentivirus were seeded into 96-well plates at a density of 3×10³ cells per well and incubated for 24 hours. Subsequently, 10μL of CCK-8 reagent (C6005, NCM Biotech) was added to each well, and the plates were further incubated for 2 hours. The absorbance was then measured at a wavelength of 450 nm.

### 2.22 EdU incorporation experiments

The EdU incorporation assay was utilized to assess cellular proliferation dynamics. EdU-labeled cells were detected and visualized following the manufacturer’s instructions, using the BeyoClick EdU Cell Proliferation Detection Kit (containing Alexa Fluor 647; Beyotime, China). The proliferation rate was determined as the percentage of EdU-positive cells relative to the total cell population.

### 2.23 Transwell migration assay

A Transwell assay was performed to assess the migratory ability of U251 cells. Following serum starvation, transfected cells were seeded into the upper chambers, while 10% fetal bovine serum (FBS) was added to the lower chambers as a chemoattractant. After incubation for 24 hours at 37°C, the migrated cells were fixed, stained, and quantified. The migration capacity was determined based on the average number of migrated cells from three randomly selected fields.

### 2.24 Statistical analysis

All tests were two-sided unless noted; data are mean ± SD from ≥3 independent experiments; significance set at P<0.05 or FDR<0.05(Benjamini-Hochberg). Experimental-normality and variance were checked (Shapiro-Wilk, Levene); two-group tests used unpaired t or Mann-Whitney; multi-group tests used one-way ANOVA with Tukey/Sidak or Kruskal-Wallis with Dunn. IHC scores used Mann-Whitney.

## 3 Results

### 3.1 Single-cell integration and annotation

**Figure 2:**
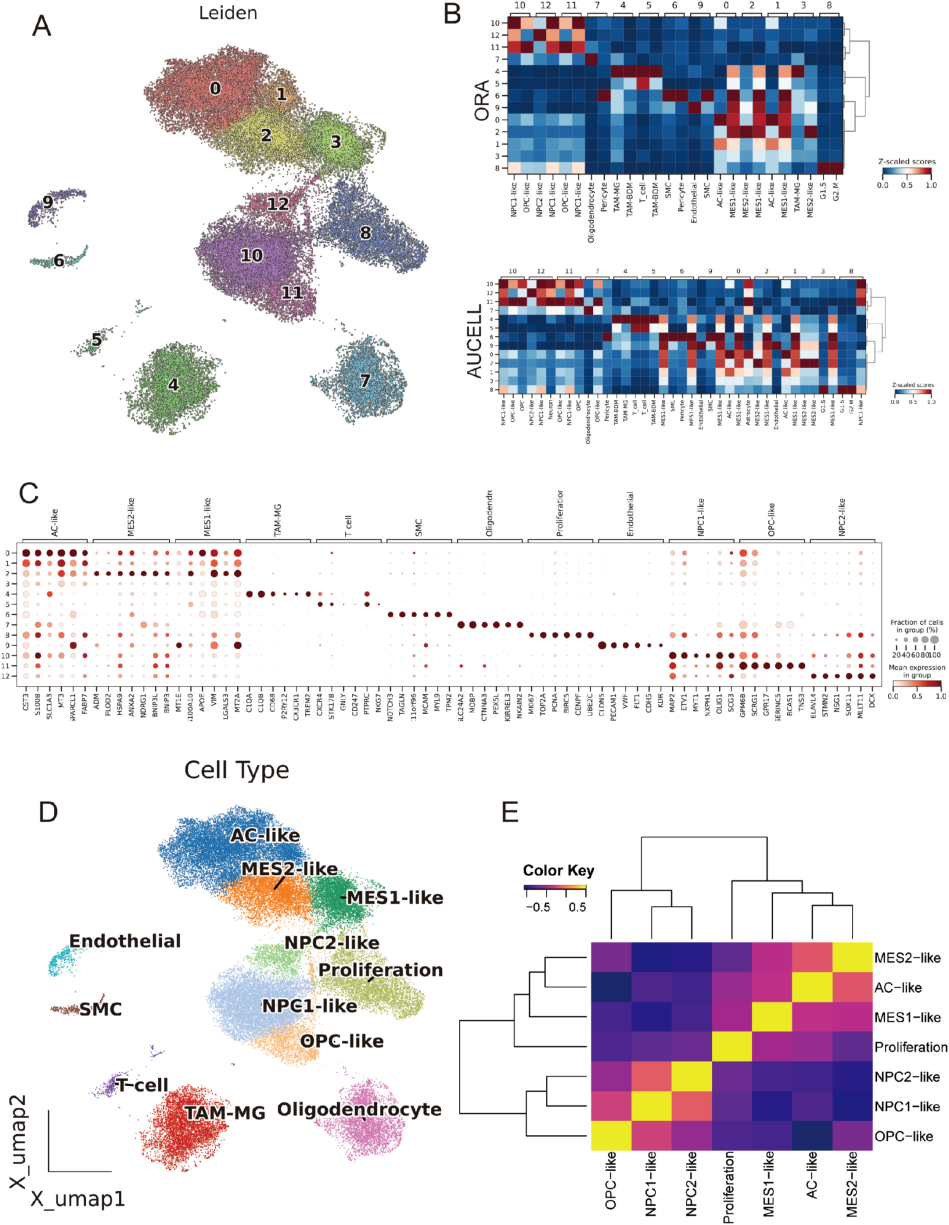
Integration, clustering and annotation of the single-cell GBM atlas. **(A)** UMAP of the Harmony-integrated dataset colored by leiden clusters (resolution =0.8; labels 0–12). **(B)** Cell-type scoring using decoupler with over-representation analysis (ORA) and AUCell enrichment. Scores are row-wise Z-scaled; columns are hierarchically clustered. **(C)** Dot plot of canonical marker genes across leiden clusters. Dot size denotes the fraction of cells expressing the gene within a cluster; color encodes mean normalized expression. **(D)** Final cell-state annotation projected onto the UMAP: AC-like, MES1-like, MES2-like, NPC1-like, NPC2-like, OPC-like, Oligodendrocyte, Proliferation, TAM-MG, Endothelial, SMC and T cell. **(E)** Pairwise correlation heatmap (Pearson’s r, Z-scaled) of cell-type signature scores.

After stringent quality control, normalization, and highly variable gene selection, we built a KNN graph in PCA space and systematically compared batch-correction strategies (ComBat, Scanorama, Harmony). Harmony best preserved biological structure while maximizing batch mixing, with an optimal resolution around 0.8 based on the joint behavior of the silhouette coefficient, LISI, and graph connectivity, yielding 12 clusters (**Figure 2A**; **Supplementary Figures 1A, B**). Cluster identities were assigned using curated marker genes together with decoupler-based ORA and AUCell scoring, recovering the major GBM-related states: astrocyte-like (AC-like); mesenchymal-like 1 (MES1-like); mesenchymal-like 2 (MES2-like); neural progenitor cell–like 1/2 (NPC1-like/NPC2-like); oligodendrocyte precursor cell–like (OPC-like); oligodendrocyte (OLG); proliferative cells (Proliferation); TAM-MG; endothelial cells (EC); and smooth muscle cells (SMC); the two scoring approaches agreed with marker-expression dot plots (**Figure 2B**). Simultaneously, using canonical markers, we annotated clusters into malignant states (MES2-like, MES1-like, NPC2-like, NPC1-like, AC-like, OPC-like, and a Proliferation-high group) and non-malignant lineages (T cells, TAM-microglia, endothelial, SMC, oligodendrocytes), which segregate in the embedding with tumor cells spanning an NPC/AC/OPC-to-MES continuum and proliferation overlaying multiple states (**Figures 2C, D**). Sample-level compositions revealed marked inter-patient heterogeneity, with several tumors enriched for MES2-like and immune populations (**Supplementary Figure 1C**). GO programs further captured cell type-specific biology-e.g., OPC enriched for myelin assembly, OPC-like cells for OPC-progenitor proliferation, TAM-MG for phagocytosis/inflammatory response, EC for sprouting angiogenesis (with blood-brain barrier maintenance), Proliferation for mitotic cell cycle/DNA replication, and MES2-like for glycolysis and hypoxia adaptation-providing a coherent functional map that mirrors the separability seen in tumor correlation patterns (**Figure 2E**; **Supplementary Figure 1D**).

### 3.2 CNV-based tumor delineation and developmental gradients across GBM states

**Figure 3:**
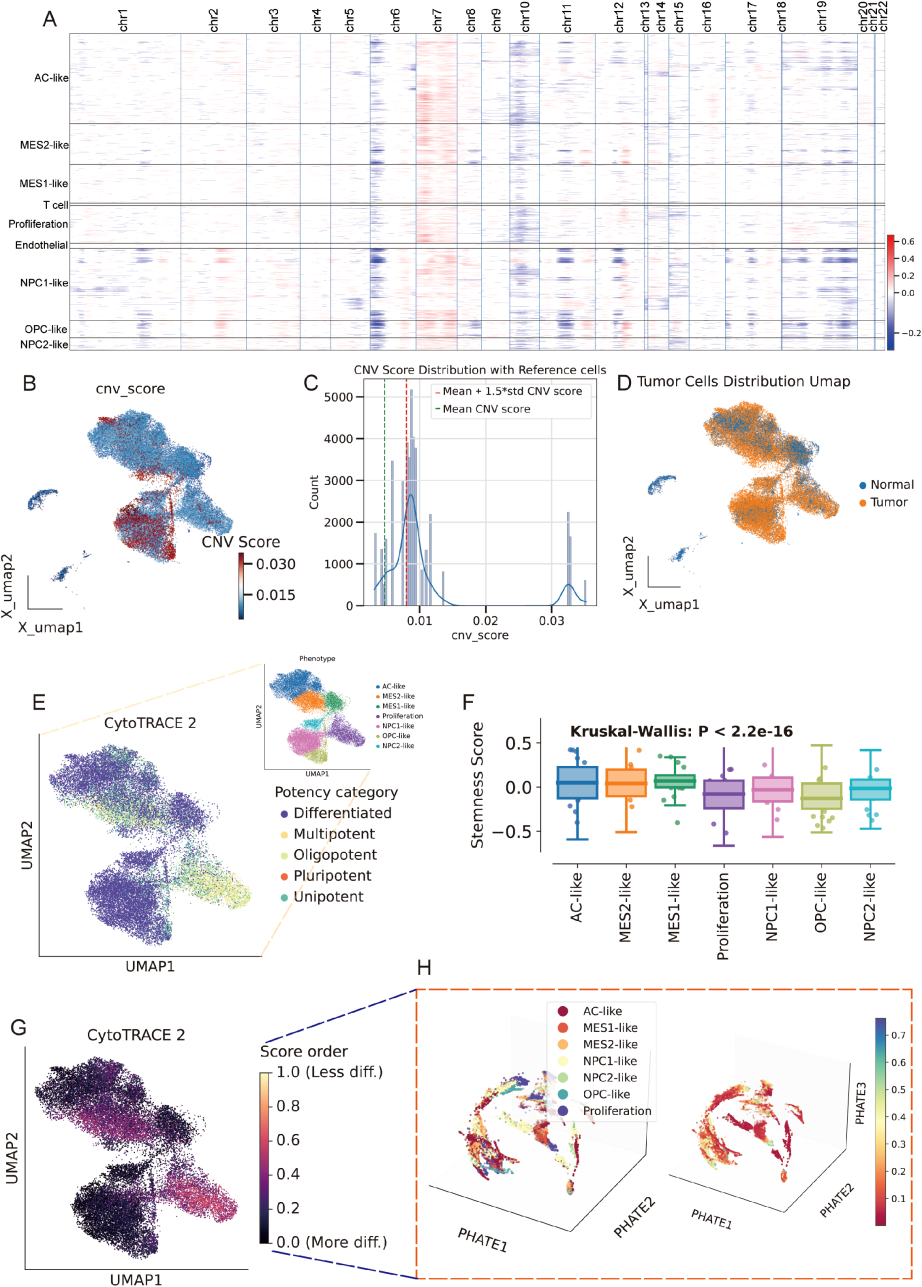
Copy-number–based malignant calling and developmental potential across GBM states. **(A)** Inferred large-scale CNVs across chromosomes (chr1–22) stratified by cell state (rows). Red/blue denote relative gains/losses. **(B)** Per-cell CNV burden projected onto the Harmony UMAP. **(C)** Distribution of cnv_score. **(D)** CNV-low (normal) and CNV-high (tumor) assignments mapped back to the UMAP. **(E)** CytoTRACE2 developmental potency projected onto the UMAP (purple = more differentiated; yellow/orange = higher potency). **(F)** Stemness scores across cell states, showing significant differences (Kruskal–Wallis P < 2.2 × 10^−16^). **(G)** CytoTRACE2 score heatmap on the UMAP highlighting continuous gradients from high-potency cores toward differentiated peripheries. **(H)** PHATE manifold colored by cell states reveals branching trajectories rather than a single linear differentiation order. *P<0.05.

Using inferCNV, we observed broad, chromosome-scale copy-number shifts across malignant clusters, with relatively flat profiles in reference lineages (EC/T cells) (**Figure 3A**). A per-cell CNV score highlighted focal regions of elevated aneuploidy on the UMAP (**Figure 3B**); setting a data-driven threshold at the reference mean + 1.5 s.d. separated putative tumor from normal cells and recapitulated their manifold distribution (**Figures 3C, D**). CytoTRACE2-based mapping of developmental potential on the unified UMAP revealed a pronounced enrichment of high-potency cells within the MES2-like and Proliferation compartments, forming a continuous outward gradient of decreasing potential. In contrast, AC-, OPC-, and NPC-like populations localized to the low-potency (differentiated) pole, consistent with more mature lineage states (**Figure 3E**; **Supplementary Table 4**). Stemness scores varied significantly across cell types (Kruskal–Wallis test, *P* < 2.2 × 10^−16^, **Figure 3F**). While group medians were broadly comparable, MES2-like cells showed a conspicuous right-skewed distribution, indicating a subpopulation with elevated CytoTRACE2-inferred stemness (**Figure 3G**). 3D PHATE embeddings did not resolve a single linear lineage; instead, multiple partially overlapping trajectories emanated from these high-stemness niches toward several differentiated endpoints (**Figure 3H**). Branches intersected and rejoin, indicating state interconversion and plasticity rather than a fixed differentiation order across tumor cell subtypes. Across malignant cell states, signaling and PTM segregated in a biologically coherent way. Across malignant states, we observed sharply delineated signaling and PTM landscapes that track with cellular phenotypes (**Supplementary Figures 2A, B**). The Proliferation state was dominated by growth-factor cascades (EGFR–PI3K–MAPK and WNT) accompanied by PTMs linked to proteome remodeling (S-nitrosylation, NEDDylation, β-hydroxybutyrylation). Lineage-biased states showed distinct axes: OPC-like cells centered on TGF-β/p53 with broad PTM reprogramming; NPC1-like cells coupled TGF-β/p53 with succinylation, malonylation, and FAT10ylation; NPC2-like cells paired TGF-β/p53 with deubiquitination, β-hydroxybutyrylation, and ATG8ylation. Mesenchymal programs bifurcated: MES2-like cells engaged hypoxia–angiogenesis and inflammatory signaling (hypoxia, VEGF, NF-κB/TNFα, JAK–STAT, with androgen inputs) and were enriched for myristoylation, S-nitrosylation, and FAT10ylation; AC-like cells emphasized p53 and NF-κB/TNFα alongside ATG8ylation, lipidation (myristoylation/palmitoylation), ISGylation, UFMylation, and malonylation. MES1-like cells featured JAK–STAT with p53/NF-κB/TNFα and PTMs such as lactylation, UFMylation, and glycosylation. Collectively, these patterns resolve tumor cell states into (i) a growth-factor–driven proliferative axis, (ii) lineage-skewed NPC/OPC programs, and (iii) stress/inflammation-dominated mesenchymal states-each with a characteristic PTM “fingerprint” that likely underpins state-specific biology.

### 3.3 Cell type-aware GNN stratifies survival and interpretable risk genes

**Figure 4:**
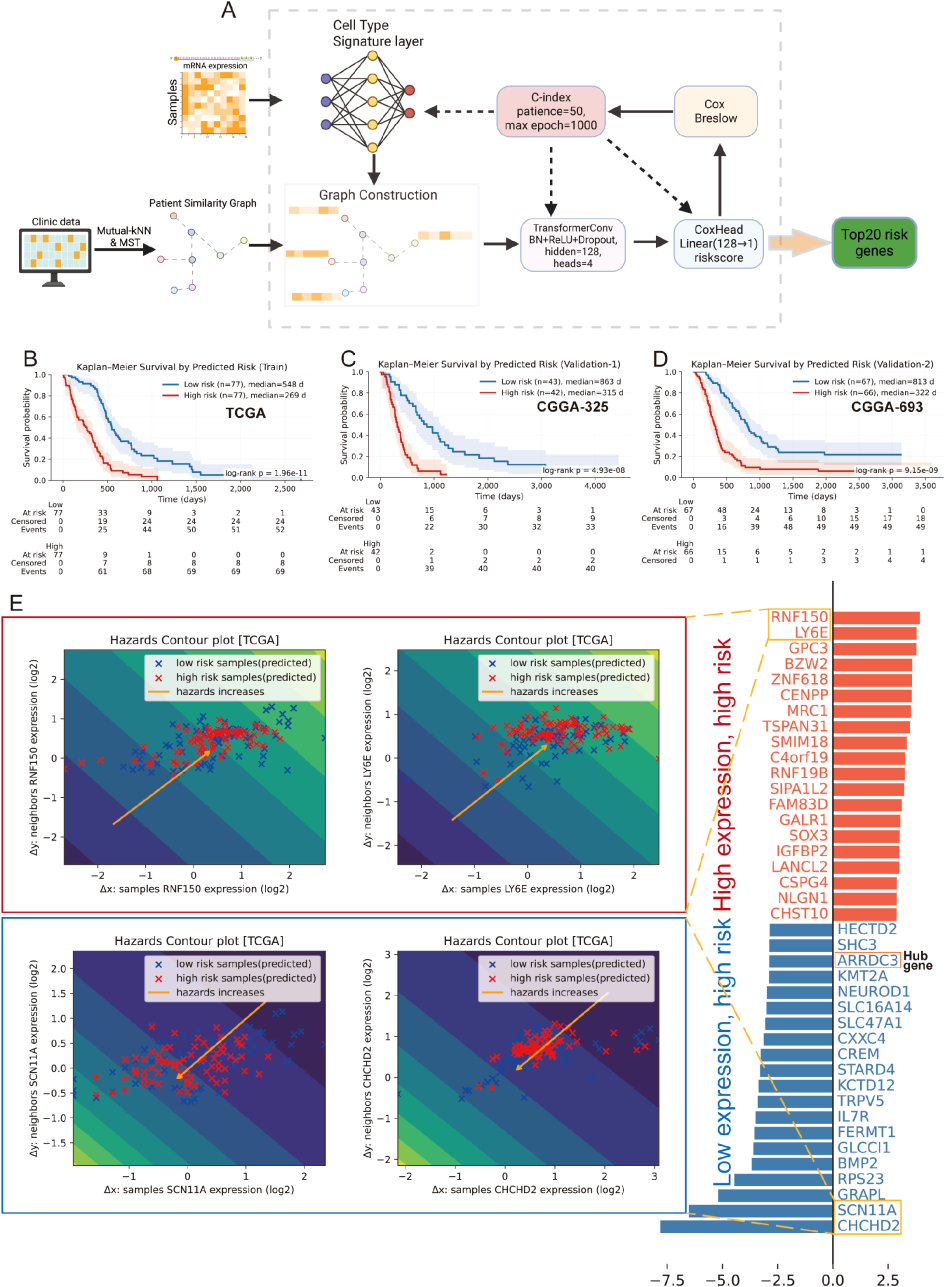
Cell type–aware graph survival modeling generalizes across cohorts and yields interpretable risk genes. **(A)** Schematic workflow of the cell type-aware, graph-based survival modeling pipeline. **(B–D)** Kaplan–Meier curves for the training set (TCGA; **B**) and two external validation cohorts (CGGA-325, **C**; CGGA-693, **D**). Samples were split by the median predicted risk. Shaded bands indicate 95% CIs; tables show numbers at risk. All splits show significant separation by log-rank test. **(E)** Left: “Hazard contour” examples illustrating how risk varies jointly with a gene’s expression in a sample (x-axis) and in its graph neighborhood (y-axis). Warmer colors indicate higher predicted hazard; yellow lines show fitted trends; points are low- (blue) and high-risk (red) patients. RNF150 and LY6E exemplify risk-enhancing genes (higher expression → higher hazard), whereas SCN11A and CHCHD2 exemplify risk associated with reduced expression. Right: ranked top-20 genes from the attribution analysis, grouped as risk-enhancing (red) versus risk-mitigating/low-expression-linked risk (blue); hub genes highlighted.

We built a cell type -constrained, graph-based survival model that integrates bulk transcriptomes with clinical similarity (**Figure 4A**, see methods). The resulting risk score separated outcomes with striking consistency. In TCGA GBM (n=157), Kaplan–Meier curves showed a clear divergence between predicted low- and high-risk groups. The two independent CGGA cohorts (CGGA-325, n=85; CGGA-693, n=140) reproduced this separation, yielding similarly steep survival gradients (**Figures 4B–D**). Thus, the graph-aware model trained on TCGA generalized without re-fitting to external data. To anatomize what drives risk, we derived gene-level attributions and summarized them as directional “hazard contour” maps that couple each gene’s expression with its neighborhood context on the patient graph. Representative RMHZ genes (high expression → high hazard), such as RNF150 and LY6E, showed monotonic increases along the hazard vector, with high-risk samples clustering in the high-expression/high-neighbor-expression quadrant. Conversely, MHZ genes (low expression → high hazard), exemplified by SCN11A and CHCHD2, displayed the opposite orientation, indicating that loss of these signals associates with poorer survival (**Figure 4E**; **Supplementary Table 5**). Aggregating across patients yielded a compact panel of top risk enhancers and protectors, providing an interpretable, network-aware signature rather than a black-box score.

### 3.4 MES2-like abundance marks an aggressive program linked to poor outcome

**Figure 5:**
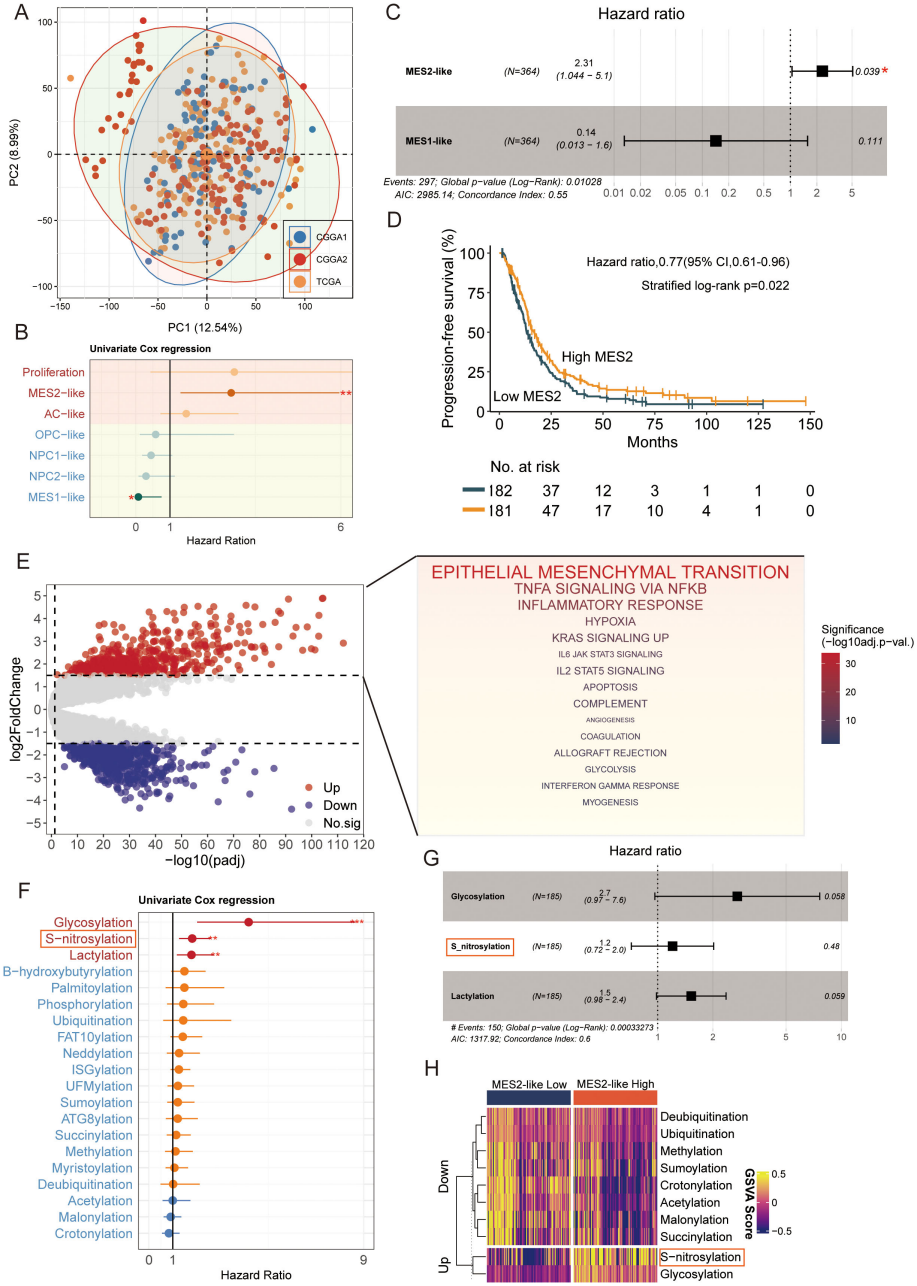
MES2-like abundance and PTM links to adverse outcome. **(A)** PCA of the pooled TCGA and CGGA cohorts after batch correction. Points are individual tumors; colored ellipses denote 95% data ellipses per cohort. Broad intermixing indicates minimal residual cohort effects. **(B)** Univariate Cox regression of malignant state fractions. Dots show hazard ratios (HRs) and bars 95% CIs. Proliferation and MES2-like associate most strongly with increased hazard; NPC/OPC/AC-like tend toward neutral/prote ctive effects. **(C)** Multivariable Cox model including malignant states. MES2-like remains an independent adverse predictor (HR = 2.31, 95% CI 1.04–5.10, P = 0.039), whereas MES1-like is not significant. The vertical dashed line marks HR = 1. **(D)** Kaplan–Meier curves for progression-free survival after stratifying tumors by MES2-like abundance (above vs. below median). The high-MES2 group shows shorter PFS (stratified log-rank P = 0.022); tables indicate numbers at risk. **(E)** Differential expression between high- vs. low-MES2 tumors. Volcano plot (left) with up-regulated genes in red and down-regulated in blue. Gene-set enrichment of up-regulated genes (right) highlights EMT, TNFα/NF-κB, hypoxia, JAK/STAT, angiogenesis, and glycolysis hallmark programs. **(F)** Outcome associations for PTM programs scored by GSVA. Univariate Cox HRs (points) with 95% CIs (bars) show that glycosylation, and to a lesser extent S-nitrosylation and lactylation, track with increased hazard, while several acylation/ubiquitylation processes trend oppositely. **(G)** Multivariable Cox model for PTM programs. **(H)** Heatmap of PTM GSVA scores (z-scaled) comparing MES2-low vs. MES2-high tumors. *P<0.05; **P<0.01; ***P<0.001.

After batch-effect correction, TCGA and CGGA samples were well intermingled in the low-dimensional embedding, indicating effective removal of cohort effects (**Figure 5A**). We then related malignant state fractions to survival (**Supplementary Table 6**). Univariate Cox models showed higher Proliferation and MES2-like fractions associated with increased hazard (**Figure 5B**). In a multivariable Cox model including significant malignant states, MES2-like remained an independent adverse predictor (HR ≈ 2.31, 95% CI 1.04–5.10, *P* = 0.039) (**Figure 5C**). Consistently, patients stratified by MES2-like abundance (above vs. below median) showed shorter progression-free survival in the high-MES2 group (stratified log-rank *P* = 0.022; **Figure 5D**). Transcriptionally, high-MES2 tumors up-regulated hallmark programs of invasion, inflammation, and metabolic stress—including epithelial–mesenchymal transition, TNFα/NF-κB signaling, hypoxia, multiple STAT axes, angiogenesis, and glycolysis (**Figure 5E**). PTM scoring further highlighted outcome-linked chemistry: in univariate Cox analyses, glycosylation (and to a lesser extent S-nitrosylation and lactylation) associated with increased hazard (**Figure 5F**); in a multivariable Cox analysis, S-nitrosylation remained significant (**Figure 5G**). Concordantly, MES2-high tumors displayed elevated glycosylation and S-nitrosylation (**Figure 5H**). This is consistent with our single-cell analyses in **Supplementary Figures 2A, B**, where MES2-like cells scored highest for hypoxia, NF-κB, and JAK/STAT pathway activity and showed enrichment of S-nitrosylation signatures. Mechanistically, hypoxia and inflammation-responsive signaling can reinforce mesenchymal transition, immune evasion, and metabolic rewiring, while S-nitrosylation, a redox-sensitive post-translational modification, fine-tunes effector proteins within these axes and has been implicated in therapy resistance. Together, these data support a model in which hypoxia, NF-κB/STAT signaling, and S-nitrosylation are key drivers of the MES2 program and likely contribute to adverse prognosis in GBM.

### 3.5 Network convergence pinpoints a robust MES2-like core program

**Figure 6:**
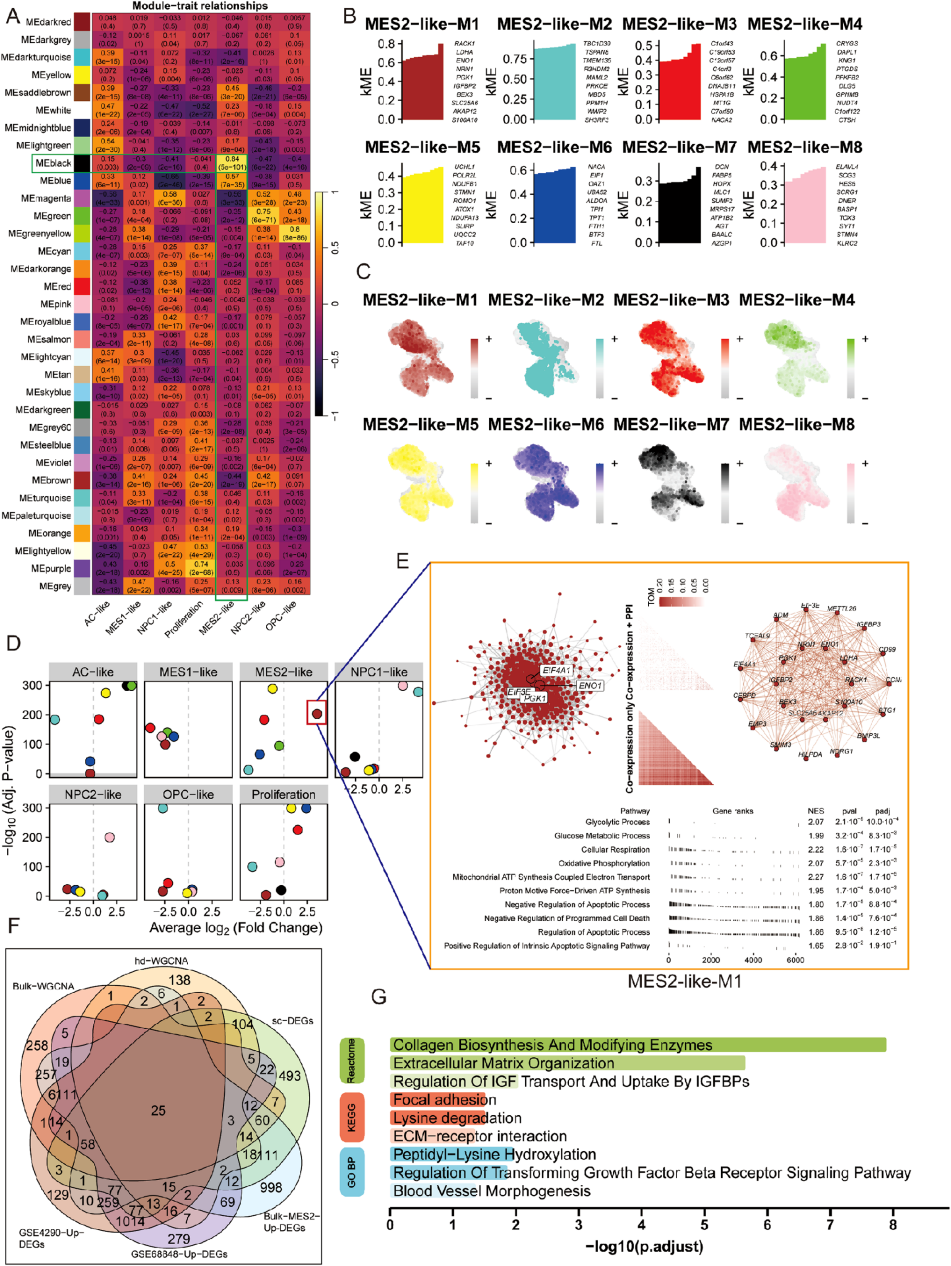
Co-expression analysis nominates a MES2-like core program. **(A)** Bulk WGCNA across TCGA/CGGA (using BayesPrism-inferred cell fractions) showing module–trait correlations. Cells display Pearson r (FDR in parentheses). Several modules—including the black module—correlate strongly and positively with the MES2-like fraction. **(B)** hdWGCNA on malignant single cells identifies eight MES2-like modules (M1–M8). Bars list representative hub genes ranked by intramodular connectivity (kME). **(C)** Feature maps of module eigengenes (hMEs) projected onto the single-cell UMAP. MES2-like modules localize to the MES2-like neighborhood. **(D)** Differential module-eigengene (DME) analysis across cell types. Points denote modules, positioned by average log_2_ fold-change (x-axis) and –log_10_ (FDR) (y-axis); MES2-like and NPC1-like–biased modules are highlighted. **(E)** Network view of the top MES2-like module (M1). Left, integrative co-expression–PPI graph (hdWGCNA TOM × STRING v12); node size reflects connectivity, labeled nodes are hubs. Right, co-expression-only layout. Bottom, fgsea of kME-ranked genes shows enrichment for glycolysis, glucose/mitochondrial respiration, apoptotic signaling, and angiogenesis programs (NES and FDR shown). **(F)** Evidence integration. Venn diagram of genes from bulk-WGCNA (TCGA/CGGA), hdWGCNA modules, MES2-like single-cell markers, bulk MES2-specific DEGs (deconvolution), and GEO validations (GSE4290/GSE68848 up-regulated). The central intersection yields a conservative MES2 core set. **(G)** Functional enrichment of the intersecting genes (GO BP/KEGG/Reactome). Top terms include collagen biosynthesis/modifying enzymes, extracellular matrix organization, focal adhesion/ECM–receptor interaction, IGFBP transport, TGF-β receptor signaling, and blood-vessel morphogenesis (bars: –log_10_; adjusted P). FDR, Benjamini–Hochberg; TOM, topological overlap matrix; kME, eigengene-based intramodular connectivity; fgsea v1.28; STRING v12.

To bridge bulk and single-cell evidence, we first performed WGCNA on bulk RNA-seq from TCGA and CGGA after deconvolution of malignant states. Several bulk co-expression modules correlated positively with the MES2-like fraction, with the black module standing out (**Figure 6A**; **Supplementary Figures 2C, D**). Genes from this black module were significantly up-regulated in the MES2-high group, and when projected to single-cell resolution, the corresponding gene-set score was selectively enriched in MES2-like malignant cells with minimal signal in other states (**Supplementary Figure 2E**). At MES2-like single cell level, hdWGCNA resolved eight MES2-like modules (M1–M8), for which intramodular connectivity (kME) highlighted recurrent hubs (e.g., collagen/ECM enzymes, redox and hypoxia-responsive genes) (**Figure 6B**; **Supplementary Figures 2F–H**). Module eigengenes localized to the MES2-like domain on the UMAP, confirming cell-state specificity (**Figure 6C**), and differential ME analyses showed selective activation of these modules in MES2-like cells relative to other malignant states (**Figure 6D**). Network visualization of the leading MES2-like module revealed tightly interlinked subnetworks enriched for an invasion-metabolic axis-glycolysis, mitochondrial respiration, apoptotic signaling, and angiogenesis (**Figure 6E**), concordant with the transcriptional hallmarks observed in high-MES2 tumors in **Figure 5E**.

We next integrated signals across modalities. Intersecting genes from bulk WGCNA (TCGA/CGGA), single-cell hdWGCNA, MES2-like single-cell markers, BayesPrism-derived bulk MES2-specific DEGs, and two independent GEO validations yielded a conservative core set, representing MES2 genes that recur across platforms and cohorts (**Figure 6F**; **Supplementary Figure 3A**, **Supplementary Table 7**). Functional enrichment of this intersects (**Figure 6G**) converged on extracellular-matrix remodeling and its regulators-collagen biosynthesis and modification, ECM-receptor interaction, focal adhesion, and TGF-b receptor signaling regulation-together with IGFBP-mediated transport and blood-vessel morphogenesis. These convergent networks position MES2-like GBM as an ECM-driven, vascular-interacting, stress-adapted state, and nominate collagen-modifying enzymes and TGF-β/adhesion nodes as tractable mechanistic anchors for the MES2 program.

### 3.6 TAM–MES2 signaling axis converges on a CEBPD-centered transcriptional program

**Figure 7:**
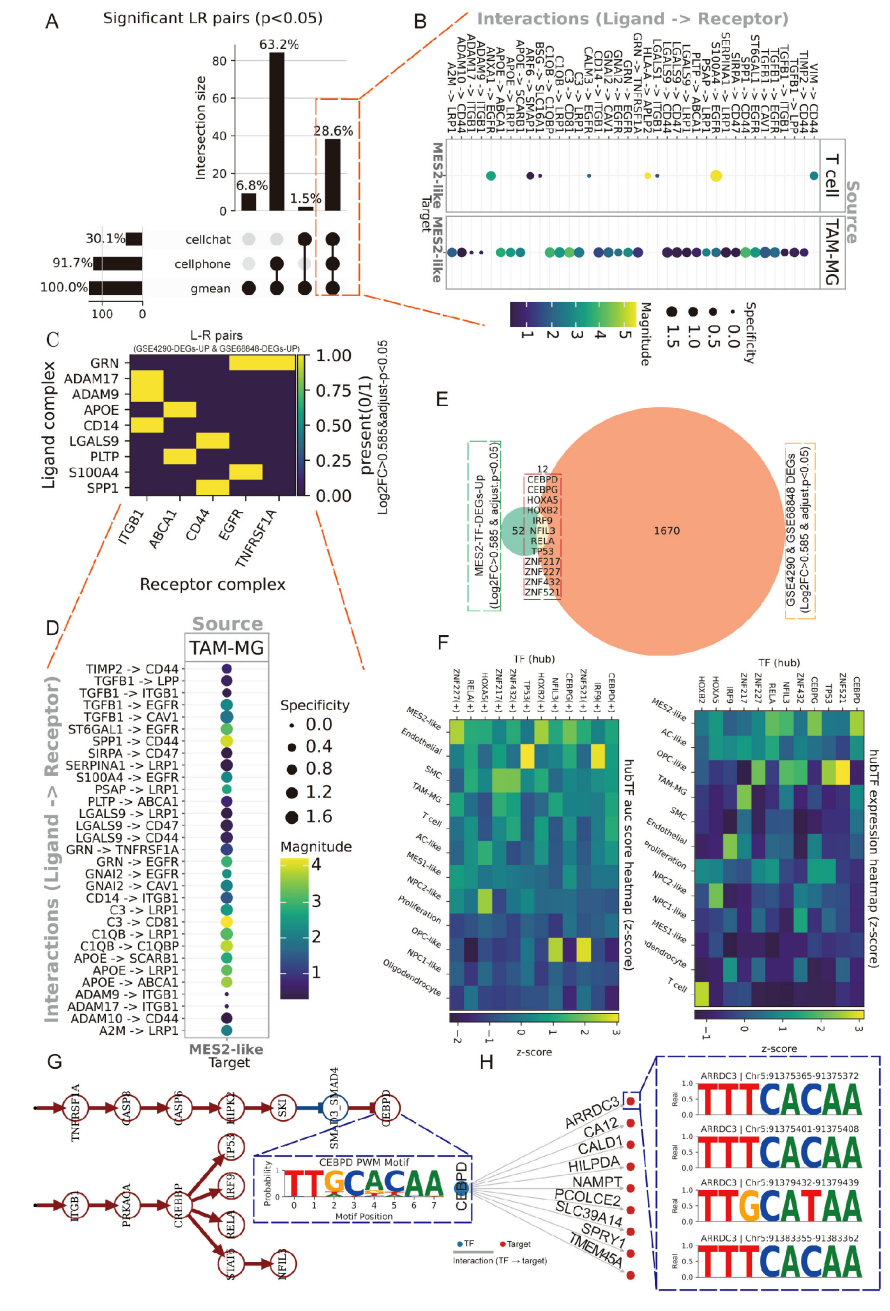
Spatially organized TAM–MES2-like communication. **(A)** UpSet summary of significant ligand–receptor (L–R) pairs (permutation P<0.05) detected by LIANA+ across three methods (geometric mean, CellPhoneDB, CellChat). Bars indicate intersection sizes; most calls were supported by all three resources. **(B)** Consensus L–R signals from immune sources toward MES2-like targets. Bubble color encodes interaction strength; size denotes specificity. **(C)** Cross-cohort validation of L–R partners. Heat map shows whether each ligand and receptor complex is up-regulated in both bulk GBM datasets (GSE4290 and GSE68848; yellow = present in both). **(D)** Ranked TAM–MG→MES2-like interactions, emphasizing specificity-enriched pairs that localize to MES2-like niches. **(E)** Intersection analysis of transcription factors (TFs) up-regulated in MES2-like cells and in GEO cohorts identifies a small, recurrent TF set; labels include CEBPD, CEBPB, HIF1A, NFKB1/2, and others. **(F)** Hub-TF activity (AUCell regulon scores, left) and expression (right) across cell states (z-scores). **(G)** Putative signaling cascade linking microenvironmental cues to MES2-like programs: TNF-receptor signaling (TNFRSF1A) converges on CEBPD, which in turn connects to MES2-hub genes. **(H)** Motif evidence for CEBPD binding in the ARRDC3 promoter (FIMO hits with representative logos), supporting a TNFRSF1A→CEBPD→ARRDC3 module at the tumor–brain interface.

We next explored the immune and MES2-like tumor microenvironment. Using LIANA+ across seven inference engines, we assembled a consensus LR atlas and retained interactions supported by multiple methods with *P* < 0.05 (**Figure 7A**; **Supplementary Figures 3C–E**, **Supplementary Table 8**). Prioritizing edges with MES2-like as the target and either TAM-MG or T cells as the source highlighted a compact set of high-magnitude, high-specificity signals (**Figure 7B**), including growth–factor pathways (TIMP2→CD44, ADAM9/10/17→ITGB1, TGFB1→ITGB1/EGFR), scavenger/clearance routes (APOE/PSAP/C1Q/C3→LRP1/ABCA1/CD81), immune–checkpoint–like axes (LGALS9/SPP1→CD44, CD47), and inflammatory cues (GRN→TNFRSF1A). We observed markedly stronger ligand–receptor signaling between TAM-MG and MES2-like cells than between T cells and MES2-like cells. Accordingly, subsequent analyses focused on the TAM-MG → MES2-like communication axis. To increase stringency, we required that both LR be up-regulated in GBM across two independent GEO cohorts (GSE4290, GSE68848); the majority of the above pairs passed this external filter (**Figures 7C, D**). At the transcriptional layer, integrative analysis of MES2-like TFs up-regulated at single-cell resolution and GBM DEGs in bulk datasets yielded a core set of candidate regulators (**Figure 7E**; **Supplementary Figure 3B**, **Supplementary Table 9**). Their regulon activity (AUC) and expression were markedly enriched in MES2-like cells (**Figure 7F**). To mechanistically connect extracellular cues to the MES2 program, we embedded the high-confidence LR inputs and MES2-enriched TFs into a signed, directed prior-knowledge network and solved for causal flows. The optimal solution converged on CEBPD as a central integrator downstream of TNFα/NF-κB, TGF-β, and MAPK/JAK–STAT cascades, linking TAM-MG-derived ligands to MES2-like transcription (**Figure 7G**). ARACNe analysis and Motif analysis and promoter scanning confirmed bona fide CEBPD binding motifs within promoters of multiple MES2-like hub genes (e.g., ARRDC3, CALD1, HILPDA, NAMPT, PLOD2, SLC39A14, SPRY1, TMEM45A), reinforcing direct regulatory control (**Figure 7H**; **Supplementary Figures 3F**, **4A**, **Supplementary Table 10**). Together, these data delineate a TAM-MG-to-MES2-like signaling axis that feeds into a CEBPD-centered transcriptional module, rationalizing how inflammatory and matrix-remodeling inputs cooperate with hypoxia/STAT/NF-κB programs to drive the MES2-like state.

### 3.7 Spatial integration, clustering, and malignant-spot calling across four GBM sections

**Figure 8:**
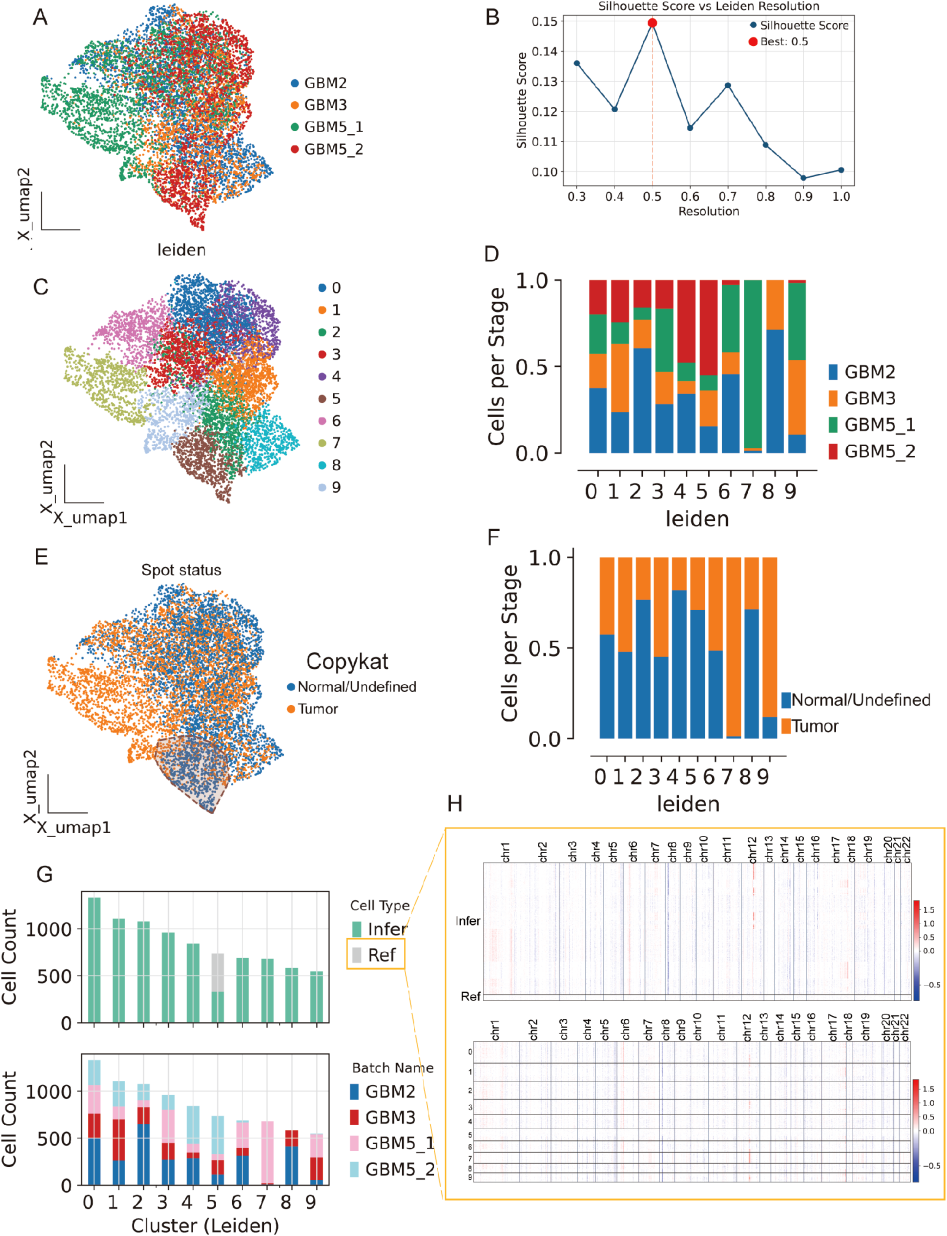
Spatial integration, clustering, and malignant-spot calling across four GBM sections. **(A)** Joint embedding (UMAP) of Visium spots from GBM2, GBM3, GBM5_1, and GBM5_2 after cross-section alignment; colors indicate specimen of origin. **(B)** Silhouette analysis over Leiden resolutions identifies a stable partition at r = 0.5 (red dot). **(C)** Leiden clusters (0–9) on the same embedding. **(D)** Cluster composition by specimen (stacked bars), showing both shared and section-specific niches. **(E)** CopyKAT large-scale CNV calls projected onto the embedding (orange, tumor/aneuploid; blue, normal/undefined). **(F)** Tumor fractions per cluster derived from CopyKAT. **(G)** Cell-count summaries: top, counts of spots labeled as Ref (reference) versus Infer (to be profiled) for inferCNV; bottom, per-cluster counts by specimen. **(H)** inferCNV heat maps across chromosomes for Infer (top) and Ref (bottom) spots, illustrating broad aneuploidy in tumor territories and diploid profiles in the reference set selected from GBM5_2.

We analyzed Visium data from GBM2, GBM3, GBM5_1, and GBM5_2. After alignment, spots from all sections were well intermixed in the low-dimensional space (**Figure 8A**), indicating effective cross-section integration. Graph-based clustering across the combined embedding yielded a stable partition at leiden resolution =0.5, which maximized the silhouette score (**Figures 8B, C**). Each cluster contained contributions from multiple sections, with sample composition varying across clusters (**Figure 8D**), consistent with both shared and specimen-specific spatial niches.

Spots from the GBM5_2 peritumoral tissue were predominantly assigned to cluster 5.

**Figure 9:**
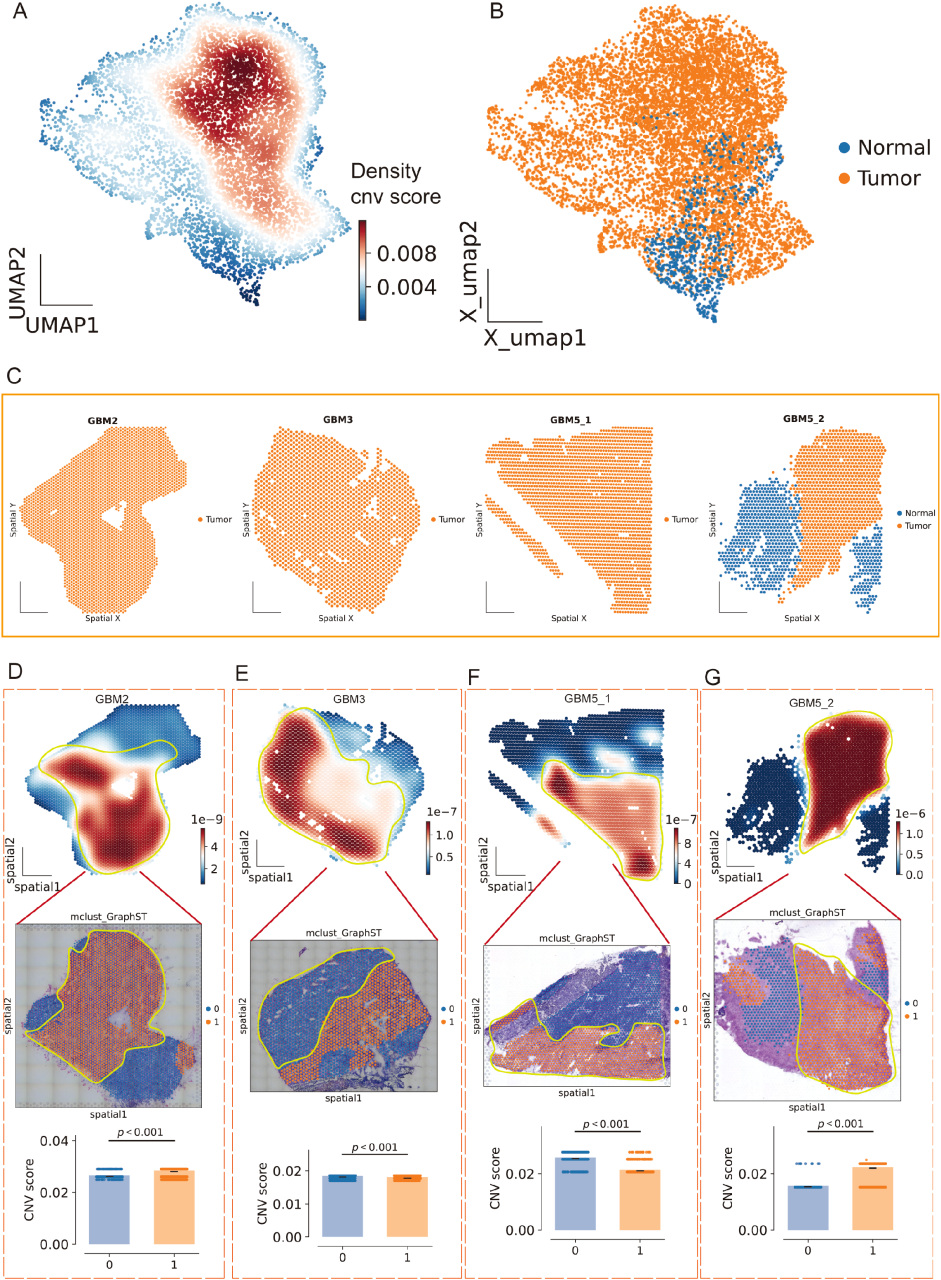
Spatial CNV gradients delineate malignant cores across GBM sections. **(A)** Joint UMAP of all Visium spots colored by local density of the continuous CNV score (inferCNV), revealing a high-aneuploidy core and tapering margins. **(B)** Tumor/normal calling on the same embedding after thresholding the CNV score using a CopyKAT-derived normal reference (mean + 2 s.d.); orange, tumor (aneuploid); blue, normal/undefined. **(C)** Tissue coordinates for each section (GBM2, GBM3, GBM5_1, GBM5_2) showing spatial localization of predicted tumor (orange) versus normal (blue) spots. **(D–G)** For each specimen, top: CNV-score heatmap over tissue (white→red, low→high) with the GraphST/mclust tumor mask outlined in yellow; middle: overlay on the matched histology image; bottom: CNV scores inside (1) versus outside (0) the mask (two-sided Wilcoxon rank-sum, p < 0.001 in all cases). CNV-high compartments co-register with computational and histopathologic tumor domains; in GBM5_2, CNV elevation extends into the peri-tumoral rim, consistent with a tumor-infiltrated normal zone.

To separate malignant from non-malignant tissue, we inferred large-scale CNV. CopyKAT classified a substantial subset of spots as aneuploid (tumor) that localized to discrete regions of the embedding (**Figure 8E**; **Supplementary Figure 4B**), and tumor fractions differed across clusters (**Figure 8F**). Concordantly, CopyKAT classified most putatively normal (aneuploid-negative) spots into cluster 5. We therefore used as the inferCNV reference only those GBM5_2 spots within cluster 5 that were labeled “normal” by CopyKAT (**Figures 8G, H**). The CNV score formed a continuous gradient with a high-aneuploidy core and tapering margins (**Figure 9A**). Thresholding the score (reference mean + 2 s.d.) yielded robust tumor vs. normal calls that reproduced this gradient (**Figure 9B**) and, when mapped back to tissue coordinates, localized to compact tumor territories in each section (**Figure 9C**). Within GBM2, GBM3, GBM5_1, and GBM5_2, CNV-high regions co-registered with GraphST/mclust tumor domains and histopathologic tumor areas (**Figures 9D–G**). Quantitatively, CNV scores were consistently elevated inside the GraphST tumor masks relative to adjacent compartments for all specimens. The high-CNV (malignant) compartments collapsed onto single dominant Leiden clusters: GBM2-cluster 1, GBM3-cluster 0, GBM5_1-cluster 1, and GBM5_2-cluster 1. Notably, in GBM5_2 the domain with elevated CNV scores spatially overlapped with the GraphST/mclust tumor cluster, indicating tumor infiltration into adjacent histologically normal tissue. We therefore annotated this region as a tumor-infiltrated normal zone. Together, these analyses reveal a spatially coherent aneuploid “malignant core” shared across patients and provide a principled scaffold for subsequent localization of MES2-like programs and cell–cell signaling.

### 3.8 Spatially localized immuno-mesenchymal signaling coincides with MES2 niches

**Figure 10:**
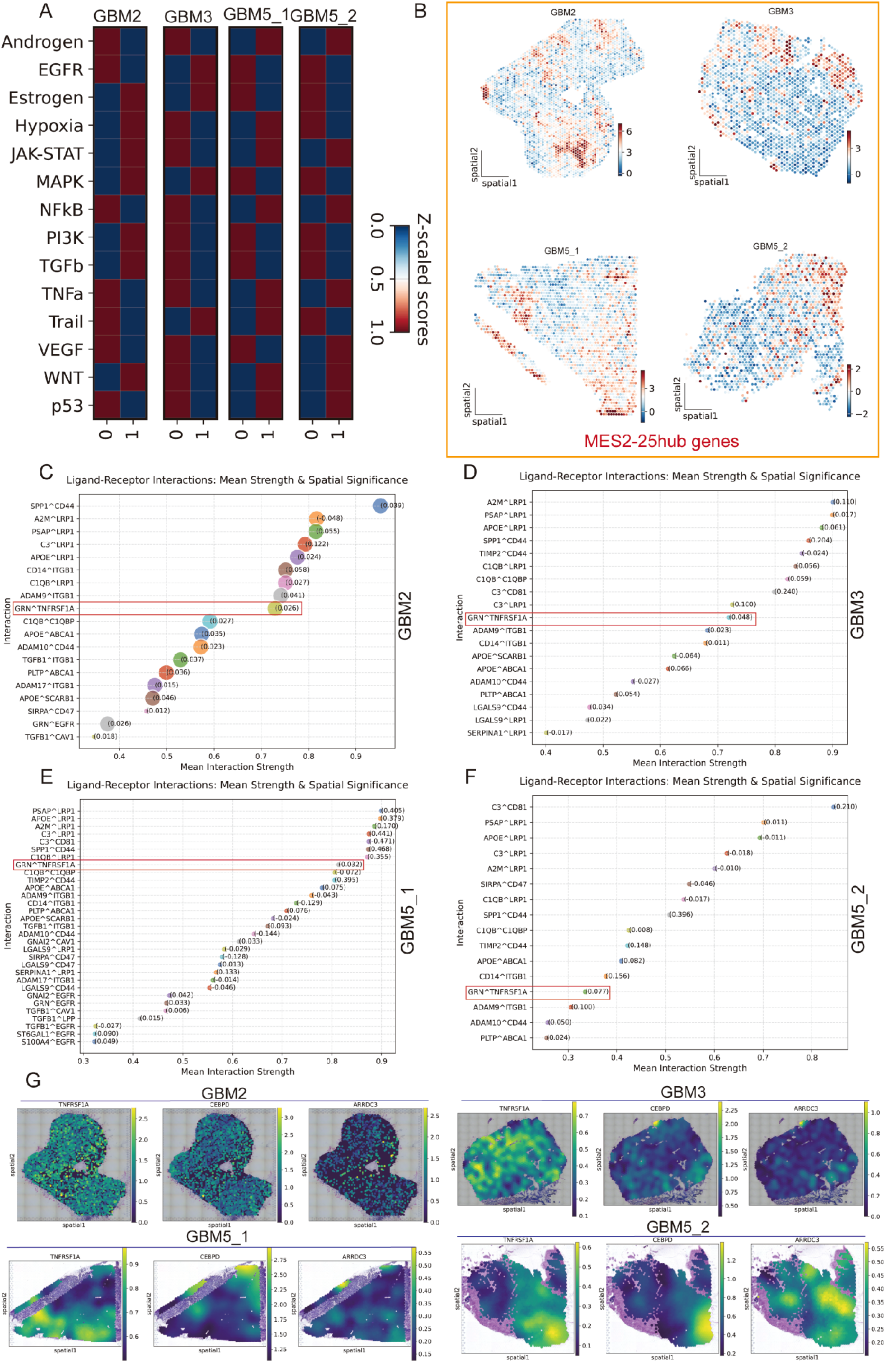
Spatial pathway activity, MES2 hubs, and TAM→MES2-like signaling converge in malignant niches. **(A)** Pathway activity (Z-scored within section) for 14 oncogenic programs across the four Visium specimens (GBM2, GBM3, GBM5_1, GBM5_2). Columns “0/1” denote cluster; tumor territories show concordant elevation of JAK/STAT and WNT, with specimen-specific features (e.g., estrogen/MAPK in GBM2). **(B)** Spatial score maps of the 25-gene MES2 hub set; warmer colors indicate higher scores. Hotspots localize within malignant regions in all sections. **(C–F)** LIANA+ (spatial mode) consensus ranking of ligand–receptor pairs from TAM-MG (source) to MES2-like (target) for each section. Points are scaled by spatial specificity (permutation-based Moran’s statistic) and colored by mean interaction strength; labels show representative top interactions. A recurrent signal is GRN→TNFRSF1A (boxed), accompanied by ADAM9/17→ITGB1, SPP1→CD44, A2M/PSAP/APOE→LRP1/ABCA1, and complement/coagulation axes, indicating an immune–mesenchymal communication hub. **(G)** Spatial expression maps for the TNFRSF1A–CEBPD–ARRDC3 axis in each section. Receptor (TNFRSF1A), transcription factor (CEBPD), and effector (ARRDC3) co-localize with MES2-hub hotspots inside tumor cores; notably, in GBM5_2 they peak at the tumor–brain interface (infiltrative rim), consistent with a spatially restricted, TAM-driven mesenchymal program.

Across all four Visium sections, spatial pathway cartography disclosed a convergent malignant program (**Figure 10A**): JAK/STAT and WNT activities were uniformly elevated within high-CNV tumor territories, whereas hypoxia signaling was tightly confined to tumor cores and conspicuously absent from the peri-tumoral infiltrative compartment of GBM5_2. Primary tumors (GBM3 and GBM5_1) shared a consistent enrichment landscape across oncogenic pathways, underscoring a common core of malignant signaling. By contrast, the recurrent specimen (GBM2) exhibited a distinct rewiring with focal enrichment of estrogen and MAPK pathways specifically within tumor regions, suggesting recurrence- or therapy-associated endocrine/mitogen-driven signaling. Together, these maps highlight (i) pan-malignant vulnerabilities (JAK/STAT, WNT), (ii) a spatial decoupling of hypoxia from infiltrative margins, and (iii) a recurrence-specific estrogen/MAPK axis that may inform stratified interventions. Spatial scoring of the MES2-hub gene set showed focal “hot spots” embedded within tumor regions of each specimen (GBM2/5_1/5_2) (**Figure 10B**). We next asked which LR signals are preferentially organized around these niches. Using LIANA+ in spatial mode with a Gaussian neighbor kernel, we aggregated consensus calls from multiple resources and ranked interactions by mean strength and spatial significance (permutation-based Moran’s P) (**Figures 10C–F**). We next focused on the two candidate receptors highlighted in **Figure 6G** (TNFRSF1A and ITGB1). Spatial maps showed that their TAM to MES2-like interactions (e.g., GRN-TNFRSF1A and CD14/ADAM9/ADAM17-ITGB1) were maximally enriched within high-CNV tumor-core domains and diminished toward infiltrative margins (**Supplementary Figures 5A–D**). We next examined the TNFRSF1A-CEBPD-ARRDC3 axis at single-spot resolution. Strikingly, receptor (TNFRSF1A), transcription factor (CEBPD), and downstream effector (ARRDC3) showed spatially co-localized high expression specifically at the infiltrative rim of GBM5\_2-the region we defined as tumor infiltration into adjacent tissue—while this coordinated peak was not observed in the other sections (**Figure 10G**). The hotspot coincided with MES2-like enrichment, elevated hypoxia/NF-κB/STAT activity, and the TAM→MES2 ligand flow (e.g., GRN→TNFRSF1A), and was supported by CEBPD motif hits in the ARRDC3 promoter. Together, these data nominate a microenvironment-linked invasion module in which TNF-receptor signaling funnels through C/EBPδ to activate ARRDC3 at the tumor-brain interface-a previously unrecognized, spatially restricted program that may underlie mesenchymal infiltration and offers a focused targetable axis.

### 3.9 ARRDC3 affects the proliferation and migration ability of U251 cells

**Figure 11:**
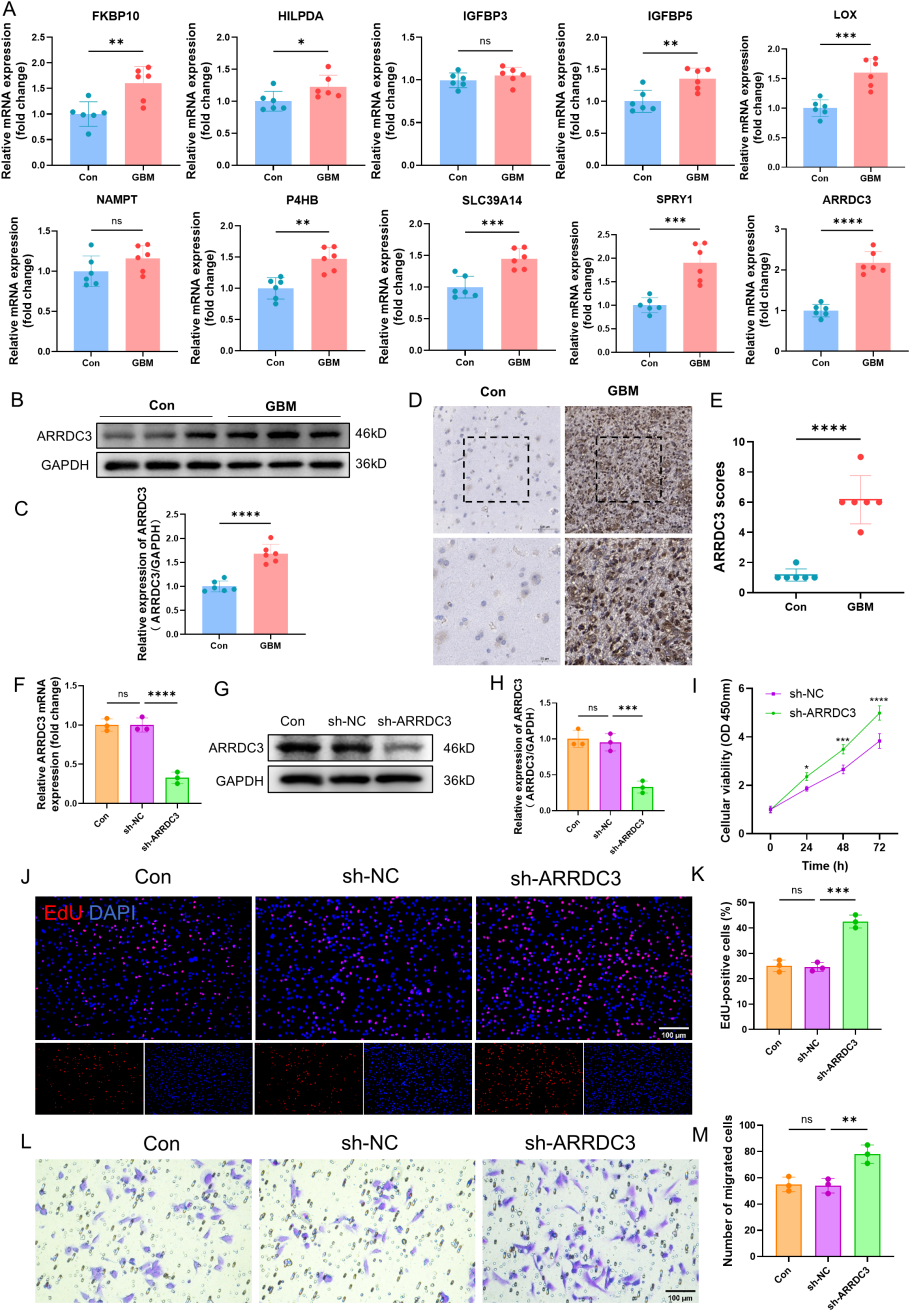
Verification of ARRDC3 expression in clinical samples and the impact of ARRDC3 knockdown on the proliferation and migration of U251 cells. **(A)** mRNA expression of differential genes in clinical samples. **(B, C)** Protein expression and quantitative results of ARRDC3 in clinical samples. **(D, E)** Representative immunohistochemical staining images and corresponding scoring results from the control group and the GBM group are presented. **(F)** mRNA expression levels of ARRDC3 in U251 cells following transfection. **(G, H)**, Protein expression and quantitative results of ARRDC3 in U251 cells following transfection. **(I)** CCK-8 reagent was added to the transfected U251 cells, and the corresponding absorbance values were measured at 24 h, 48 h, and 72 h at a wavelength of 450 nm. **(J, K)** EdU incorporation assay assessing U251 cell proliferation, and quantitative analysis of EdU-positive cells relative to total DAPI-stained cells. **(L, M)** The transwell migration assay was performed, and quantitative analysis was conducted to assess the number of migrated U251 cells. *P<0.05, **P<0.01, ***P<0.001, ****P<0.0001. ns, no significance.

Firstly, we validated the mRNA expression levels of several previously identified DEGs in both the control group and GBM group (**Figure 11A**). Among these genes, ARRDC3 mRNA was markedly upregulated in the GBM group (*P* < 0.0001). Furthermore, the protein expression level (*P* < 0.0001) and immunohistochemical scoring results (*P* < 0.0001) of ARRDC3 were also significantly elevated in the GBM group compared to the control group (**Figures 11B–E**).

To investigate the functional role of ARRDC3, we performed lentivirus-mediated knockdown of ARRDC3 in U251 cells. Both the mRNA (*P* < 0.0001) and protein levels (*P* < 0.001) of ARRDC3 were significantly reduced following transfection (**Figures 11F–H**). Cell viability assays using CCK-8 demonstrated that the viability of U251 cells in the sh-ARRDC3 group was significantly higher than that in the sh-NC group at 24 h (*P* < 0.05), 48 h (*P* < 0.001), and 72 h (*P* < 0.0001) (**Figure 11I**). The EdU incorporation assay further confirmed that the proliferative capacity of U251 cells was enhanced after ARRDC3 knockdown (*P* < 0.001) (**Figures 11J, K**). Moreover, Transwell assay results indicated a significant increase in the migratory ability of U251 cells following ARRDC3 knockdown (*P* < 0.01) (**Figures 11L, M**).

To further substantiate these findings, we conducted gain-of-function experiments by overexpressing ARRDC3 in U251 cells. The mRNA (*P* < 0.01) and protein levels (*P* < 0.001) of ARRDC3 were significantly increased after transfection (**Supplementary Figures 6A–C**). Notably, the cell viability of the OE-ARRDC3 group was significantly lower than that of the OE-NC group at 24 h (*P* < 0.05), 48 h (*P* < 0.001), and 72 h (*P* < 0.0001) (**Supplementary Figure 6D**). Consistently, both the proliferative capacity (*P* < 0.0001) and migratory ability (*P* < 0.01) of U251 cells were significantly suppressed upon ARRDC3 overexpression (**Supplementary Figures 6E–H**).

## 4 Discussion

Using integrated single-cell, bulk, and spatial transcriptomics, we delineate a mesenchymal-like (MES2-like) malignant state in GBM as a central driver of aggressiveness, therapeutic resistance, and poor prognosis, consistent with emerging evidence that MES-like transitions couple hypoxia-induced metabolic rewiring to immune evasion (33, 34). MES2-like abundance independently predicts shorter survival after adjustment for other malignant states and is marked by enrichment of epithelial-to-mesenchymal transition, NF-κB/TNFα and JAK/STAT signaling, angiogenesis, glycolysis, and hypoxia-adaptation programs; These observations suggest that MES2-like features may serve as a contextual biomarker to stratify patients and to prioritize combinational strategies (e.g., myeloid-reprogramming with checkpoint blockade, or hypoxia/metabolic-axis targeting) rather than checkpoint inhibition alone. We therefore frame MES2-like signatures as hypothesis-generating predictors whose clinical utility must be established in prospective, independent cohorts with harmonized sampling and endpoints. These features accord with reports that MES-like GBM increases glucose consumption under hypoxic stress, fostering invasion and resistance to temozolomide and radiotherapy (35). Furthermore, MES2-like states have been repeatedly linked to an immunosuppressive, myeloid-enriched microenvironment and to inferior responses to standard therapies in GBM. Prior single-cell and integrative spatial studies map MES1/MES2 programs to hypoxic niches with heightened myeloid infiltration and NF-κB/TNF pathway activity, features associated with T-cell dysfunction and treatment resistance. These observations suggest that MES2-like features may serve as a contextual biomarker to stratify patients and to prioritize combinational strategies (e.g., myeloid-reprogramming with checkpoint blockade, or hypoxia/metabolic-axis targeting) rather than checkpoint inhibition alone. We therefore frame MES2-like signatures as hypothesis-generating predictors whose clinical utility must be established in prospective, independent cohorts with harmonized sampling and endpoints (36–38) Post-translational modification profiling further implicates S-nitrosylation and glycosylation as outcome-linked hallmarks in MES2-high tumors, indicating a redox- and glycan-sensitive regulatory axis that remodels the proteome, modulates antitumor immunity, and sustains therapy refractoriness. To translate these signals clinically, we developed a cell type aware graph neural network that integrates patient similarity with transcriptomic priors and outperforms conventional models while yielding an interpretable, network-aware risk signature. Within this framework, ARRDC3 emerges as a low-expression, high-risk gene. Prior studies describe ARRDC3 as a tumor suppressor in other cancers, where it attenuates GPCR signaling and limits invasion, and additional evidence links ARRDC3 polymorphisms and expression to glioma susceptibility and adverse outcomes in related malignancies (39, 40). Together with our data, these observations support a putative CEBPD-ARRDC3 regulatory axis that interfaces with MES2-like programs to may promote immune evasion and treatment resistance. Nevertheless, in this study the axis is inferred-from single-cell and spatial expression patterns, transcription-factor activity scoring, and cis-element enrichment-rather than demonstrated by direct binding or causal perturbation. Although our preliminary analyses of ARRDC3 align with the predicted direction of effect, these findings remain exploratory and do not exclude parallel regulation or context-dependent mechanisms.

Central to this MES2 program is a TAM-MG-driven signaling axis that converges on the transcription factor CEBPD, integrating extracellular cues from ligands like GRN, APOE, and TGFB1 to orchestrate downstream effectors such as TNFRSF1A and CEBPD. This pathway, supported by consensus L-R-TFs inference and causal network modeling, highlights CEBPD as a master regulator of hypoxia-regulated invasion and stemness in GBM, corroborating studies where CEBPD augments extracellular matrix-integrin interactions and promotes glioma stem-like cell maintenance, thereby enhancing TMZ resistance (41, 42). Our data further implicate TAM-MG as key inducers of MES-like transitions via lipid-mediated metabolic interplay and M2-polarizing ligands, extending observations that macrophages reprogram GBM cells toward mesenchymal states through reciprocal interactions that sustain tumor progression and immunosuppression (7, 43).

Our integrated spatial transcriptomic analysis of four GBM sections provides a framework for dissecting the molecular and spatial heterogeneity of this aggressive tumor. Joint analysis of Visium data from primary (GBM3, GBM5_1), recurrent (GBM2), and peritumoral (GBM5_2) specimens achieved robust cross-section alignment and clustering, revealing shared and specimen-specific niches (44). These findings extend prior studies on GBM intratumoral diversity by offering a multi-patient view of conserved tumor structures (45). A major contribution is the CNV-based separation of malignant and non-malignant areas using CopyKAT and inferCNV, yielding gradient scores that align with histopathological and GraphST domains. In GBM5_2, CNV elevation in histologically normal tissue defined as a “tumor-infiltrated zone,” supporting evidence of microscopic infiltration driving high recurrence rates. This approach could enhance surgical precision and residual disease monitoring in spatial omics (46). Spatial pathway analysis highlighted pan-malignant JAK/STAT and WNT activation in tumor territories, marking them as therapeutic targets for proliferation and stemness (47, 48). Hypoxia was restricted to cores, suggesting normoxic invasion drivers warranting model validation (49). Recurrent GBM2 showed distinct estrogen and MAPK enrichment, implying therapy-related adaptations and stratified treatments (50, 51). MES2-like hotspots in tumors linked to TAM-MES2 interactions via TNFRSF1A and ITGB1. A TNFRSF1A-CEBPD-ARRDC3 axis peaked at GBM5_2’s infiltrative rim, co-localizing with MES2, hypoxia/NF-κB/STAT, and GRN signaling, proposing a novel C/EBPδ-mediated invasion module (52). This context-specific axis offers targeted opportunities, like TNFRSF1A or CEBPD inhibition, to block mesenchymal invasion without impacting core tumor pathways. Our findings build upon the mesenchymal axis previously described by Xiao et al., through the biochemical subdivision of the MES subtype into distinct molecular programs defined by PTMs (7). the establishment of EPAS1-dominant chronic hypoxia as a hallmark at the regulon level; the identification of a TAM-to-MES2 ligand-receptor signaling funnel that converges on a CEBPD-centered regulatory module; the spatial localization of MES2 within genomic regions enriched for CNVs; and the demonstration of independent and generalizable prognostic significance. Together, these advances underscore the TNFRSF1A/ITGB1/EGFR–CEBPD signaling axis and specific PTM processes—particularly S-nitrosylation—as promising, therapeutically actionable targets in MES2-like GBM.

This retrospective, multi-platform design (scRNA-seq, bulk, Visium) may retain batch/sampling bias; This study is limited by sample size, patient composition, and treatment timeframe. Certain cell subsets may be underrepresented. These conclusions require further validation in a larger, multicenter cohort encompassing both relapse and post-treatment timeframes. Future work should include proteomic/spatial validation, targeted perturbations, and multi-center longitudinal cohorts with prospective clinical calibration, and risk-stratified immunotherapy will be explored.

## 5 Conclusion

We integrated single-cell, bulk, and spatial transcriptomics with CNV profiling and a cell type–aware GNN to define a robust MES2-like program that maps to high-CNV cores, shows elevated stemness, and independently predicts poor outcome (HR = 2.31); the GNN score generalized across TCGA/CGGA. PTM analyses link S-nitrosylation and glycosylation to MES2-associated hypoxia/inflammation. A TAM→MES2 axis (GRN→TNFRSF1A; ADAM9/10/17→ITGB1; TGFB1→ITGB1/EGFR) converges on CEBPD, with a TNFRSF1A–CEBPD–ARRDC3 hotspot at the infiltrative rim; in U251 cells, ARRDC3 suppresses proliferation/migration when overexpressed and promotes them when silenced. Translationally, combine the MES2 fraction and GNN score for stratification, prioritize CEBPD and TNFRSF1A/ITGB1 (with potential hypoxia/NF-κB/JAK–STAT co-inhibition), and target infiltrative rims.

## Data Availability

The original contributions presented in the study are included in the article/**Supplementary Material**. Further inquiries can be directed to the corresponding authors.
