# Supplementary material for: Single-cell/spatial integration reveals an MES2-like glioblastoma program orchestrated by immune communication and regulatory networks
Source: Front Immunol. 2025 Oct 29;16:1699134. doi: 10.3389/fimmu.2025.1699134 (PMC12604987; doi:10.3389/fimmu.2025.1699134)
Supplement: Supplementary file 1 [file Table1.docx]

**Supplementary Figure 1. Cross-sample integration, clustering selection, and functional annotation of the single-cell GBM atlas.**


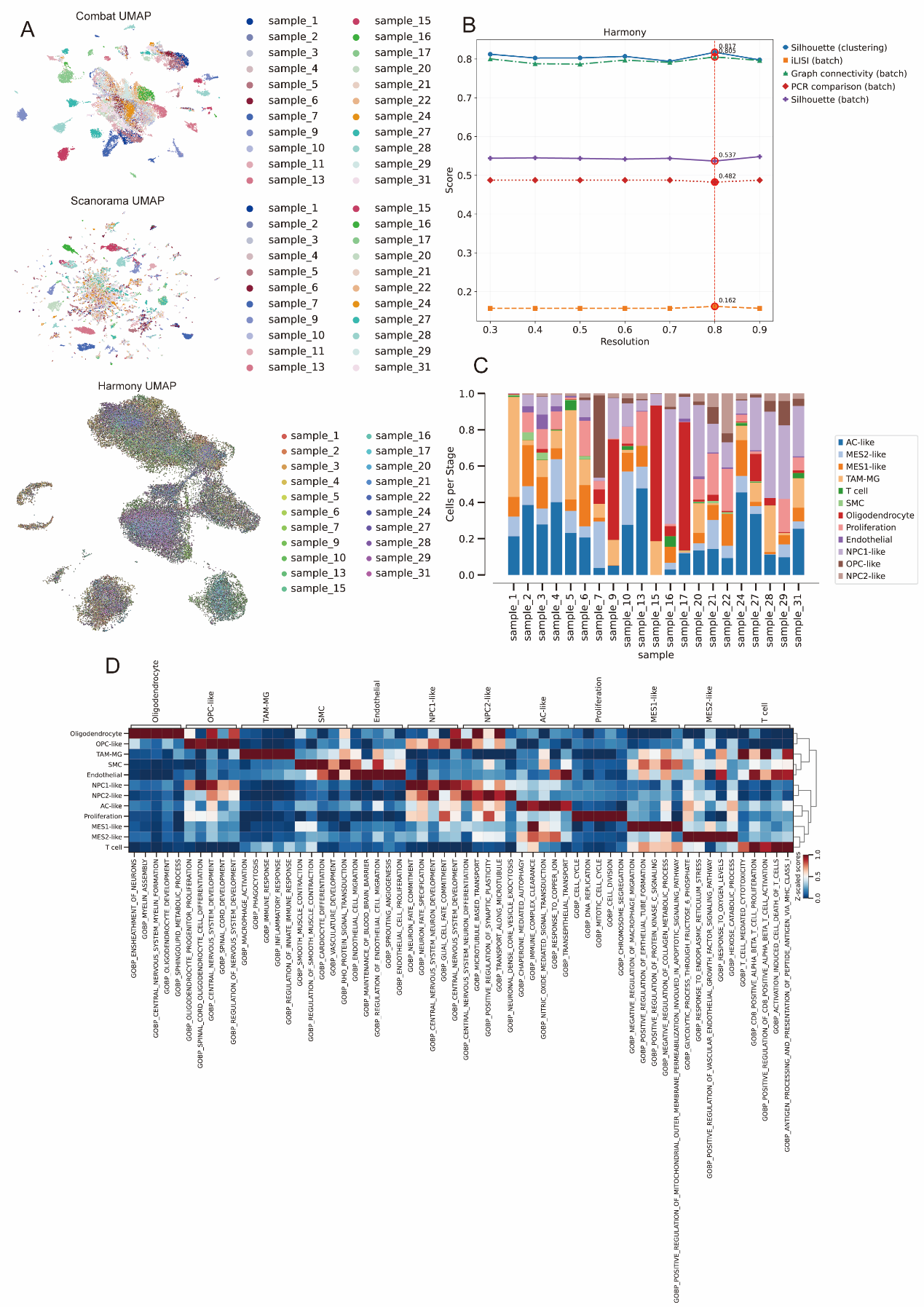


**Supplementary Figure 1. Cross-sample integration, clustering selection, and functional annotation of the single-cell GBM atlas.**

A, UMAP embeddings after batch correction with ComBat, Scanorama, and Harmony, colored by sample. Harmony yields the best cross-sample mixing while preserving biological structure.

B, Integration/clustering diagnostics across Leiden resolutions for the Harmony embedding. Curves show clustering Silhouette (teal), batch LISI (green; higher is better mixing), graph connectivity (purple), and PCR comparison for batch (pink), with batch Silhouette (orange). The vertical dashed line marks the chosen resolution (=0.8), which maximizes clustering Silhouette while maintaining strong batch mixing and connectivity.

C, Stacked bar plots of cell-type composition per sample following annotation (AC-like, MES1/2-like, NPC1/2-like, OPC-like, Oligodendrocyte, Proliferation, TAM-MG, Endothelial, SMC, T cell), highlighting inter-individual heterogeneity.

D, Heatmap of pathway activities (Z-scored) per cell type derived from GO Biological Process gene sets (MSigDB) using decoupler.

**Supplementary Figure 2. Pathway/PTM activity maps and co-expression network construction/quality assessment.**
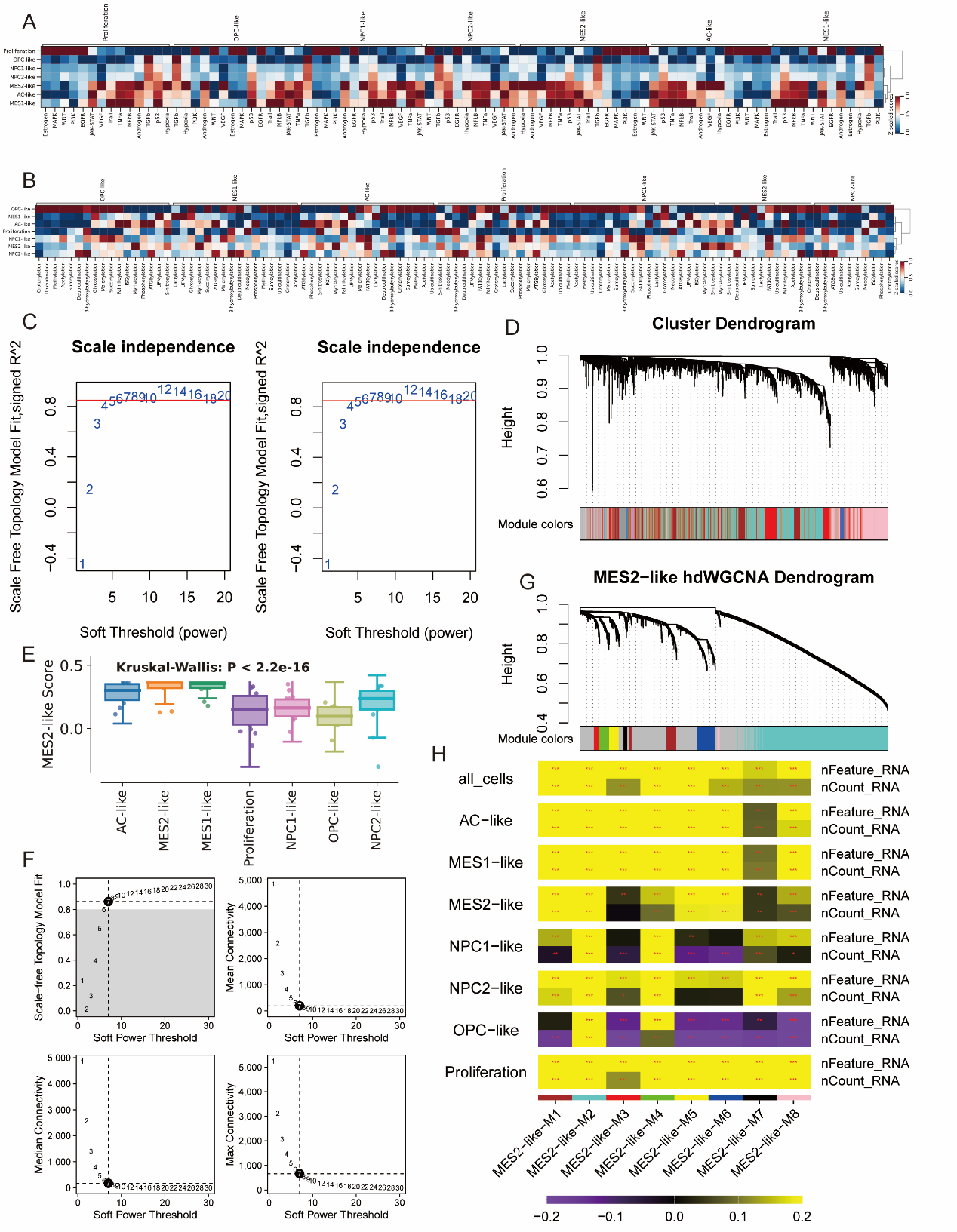


**Supplementary Figure 2. Pathway/PTM activity maps and co-expression network construction/quality assessment**

A–B, Z-scored activities of post-translational modification (PTM) programs (A) and oncogenic signaling pathways (B) across annotated malignant states.

C, Soft-threshold selection for bulk WGCNA using the scale-free topology criterion;

D, Bulk WGCNA gene dendrogram with dynamic tree-cut module assignment (color bar).

E, Distribution of MES2-like signature scores across cell states (Kruskal–Wallis P < 2.2×10⁻¹⁶).

F, Additional network diagnostics for hdWGCNA: scale-free model fit and mean/median/max connectivity versus soft-power; vertical dashed lines indicate selected power.

G, hd WGCNA gene dendrogram with dynamic tree-cut module assignment (color bar).

H, Module–technical trait correlations across cell types.

**Supplementary Figure 3. Identification of MES2-like hub genes and convergent ligand–receptor signaling from TAM-MG to MES2**
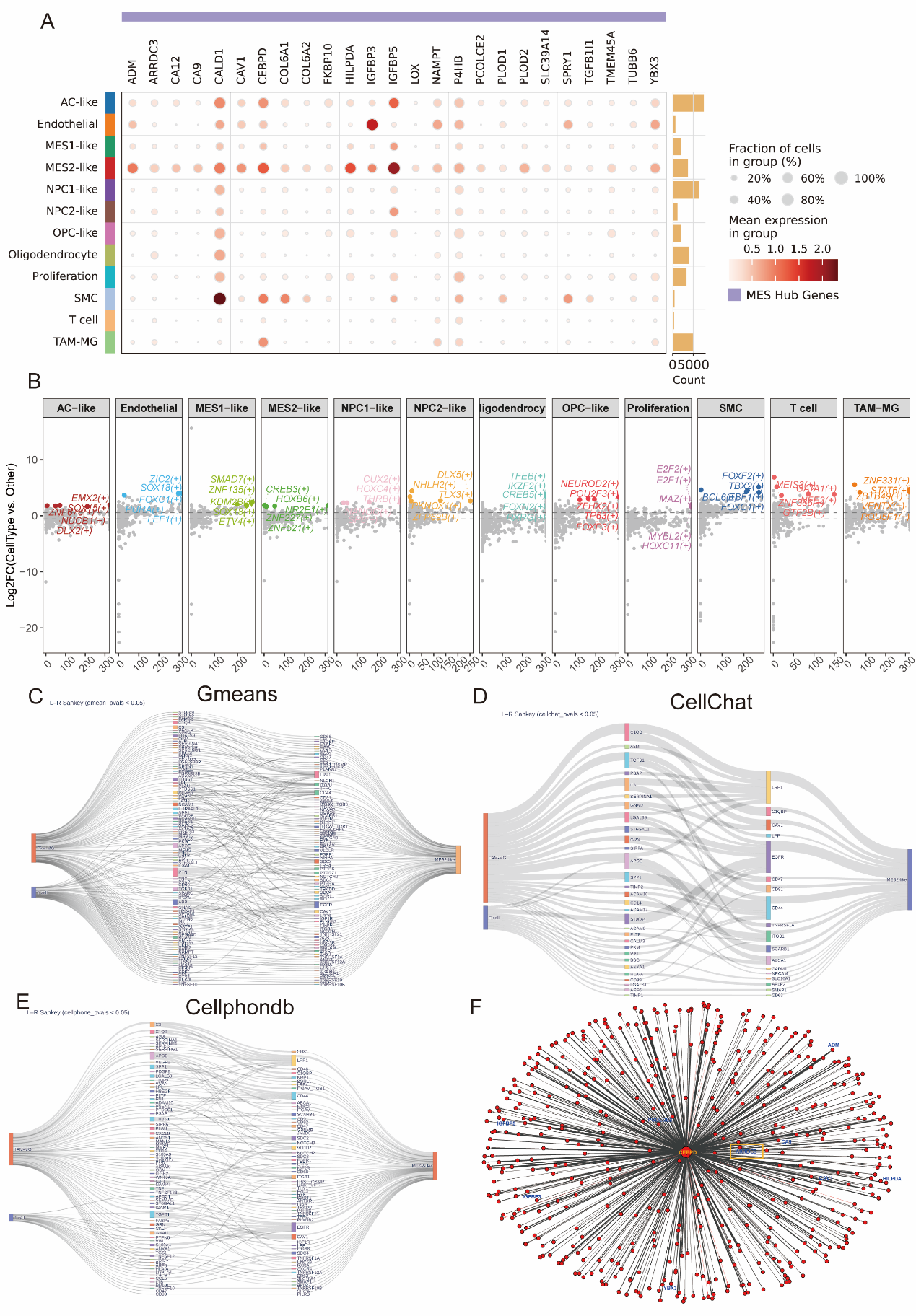


**Supplementary Figure 3. Identification of MES2-like hub genes and convergent ligand–receptor signaling from TAM-MG to MES2**

A, Dot plot of MES2-hub genes across cell states. Average expression (color) and fraction of expressing cells (dot size) for the curated MES2-like hub set. Bars at right, per–cell-type spot counts. MES2-like cells show the dominant enrichment.

B, Cell-type–resolved differential expression of the hub set. For each cell state (facets), log₂ fold-change (vs. all other states) is shown for hub genes (gray dots; exemplar genes annotated), confirming selective up-regulation in MES2-like tumor cells and relative depletion in non-mesenchymal states.

C–E, Consensus spatial ligand–receptor signaling toward MES2-like cells. Sankey plots summarize significant interactions (edge width ∝ interaction strength/significance, P<0.05) identified by three complementary LIANA+ resources: (C) geometric-mean (gmeans), (D) CellChat, and (E) CellPhoneDB. Sources (left) include TAM–MG and T cells; targets (right) are MES2-like cells.

F, Integrated L–R–TF–target network focused on MES2-like regulation.

**Supplementary Figure 4. C/EBPδ motif support at MES2-hub promoters and copy-number–linked transcriptional shifts**


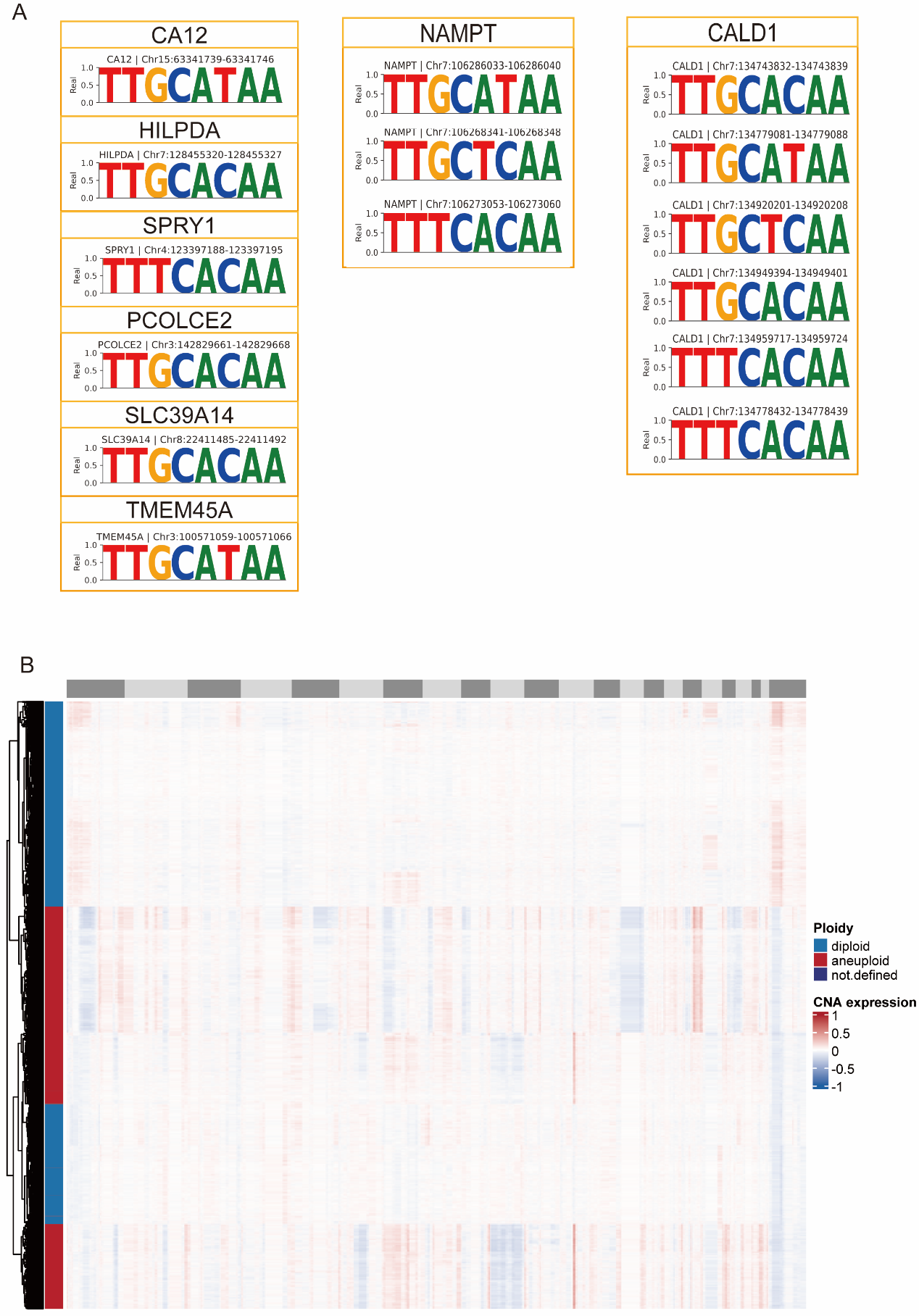


**Supplementary Figure 4. C/EBPδ motif support at MES2-hub promoters and copy-number–linked transcriptional shifts**

A, Promoter‐level CEBPD motifs in MES2-hub genes. Representative FIMO hits (JASPAR-2024 C/EBPδ PWM) within −1 kb/+100 bp of the TSS for selected MES2-hub genes (CA12, NAMPT, CALD1, HILPDA, SPRY1, PCOLCE2, SLC39A14, and TMEM45A). For each gene, the sequence logo and the genomic coordinate of the motif instance are shown.

B, Copy-number–associated expression across spatial spots. Heat map of CNA-expression scores (row-wise scaled; blue, decreased; red, increased) for Visium spots, ordered by genomic position. Left color bar denotes ploidy class inferred by CopyKAT (blue, diploid; red, aneuploid; grey, not defined). Aneuploid spots exhibit coherent, chromosome-scale expression deviations consistent with large-scale CNAs, validating malignant–normal separation used in downstream spatial analyses.

**Supplementary Figure 5. Spatial maps of TAM→MES2 ligand–receptor signaling across GBM sections**
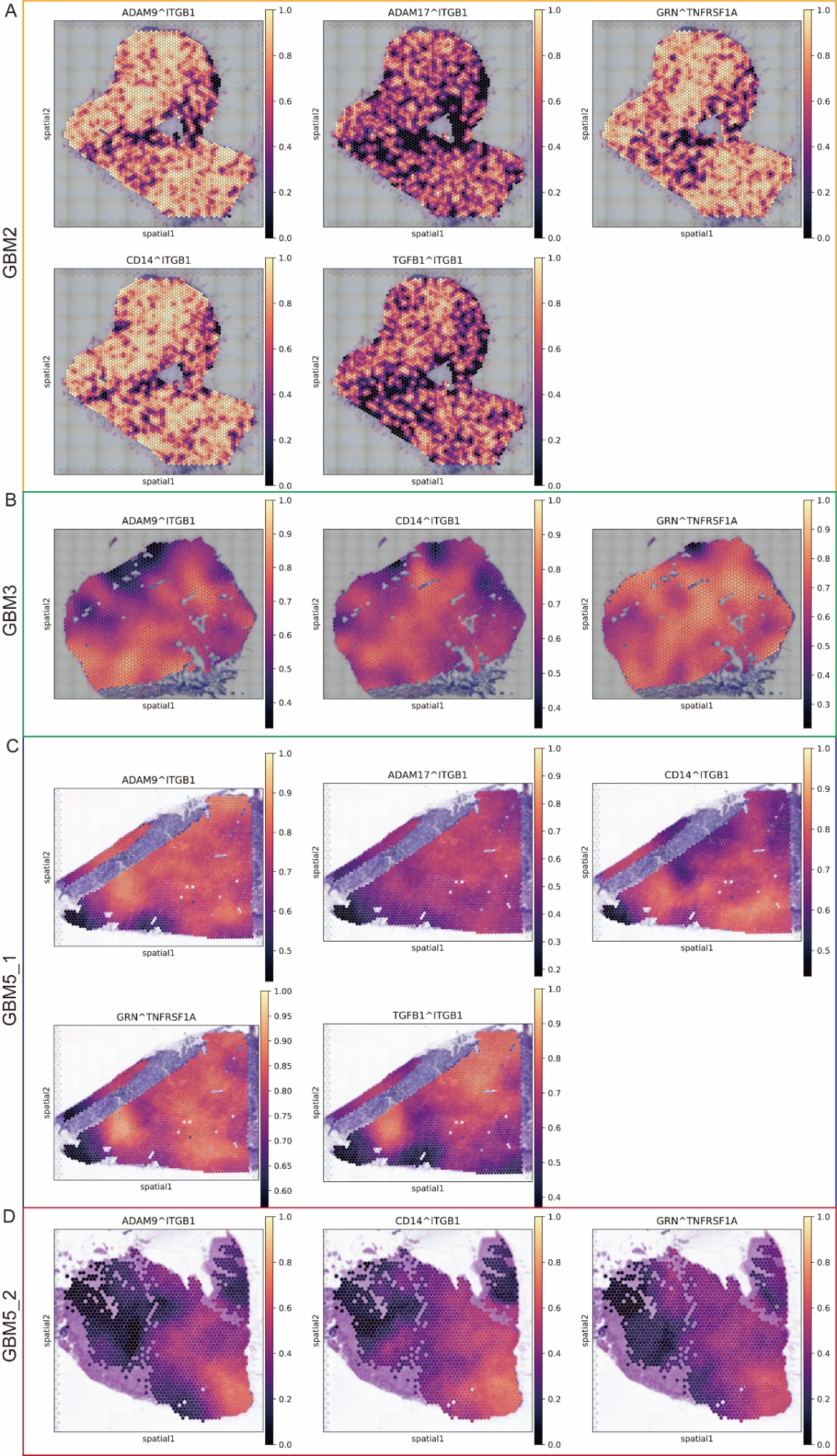


**Supplementary Figure 5. Spatial maps of TAM→MES2 ligand–receptor signaling across GBM sections**

A–D, GBM2, GBM3, GBM5_1 and GBM5_2. Visium maps show LIANA+ spatial interaction scores (0–1, color bar) for TAM-derived ligands engaging MES2-like receptors: ADAM9→ITGB1, ADAM17→ITGB1, CD14→ITGB1, TGFB1→ITGB1, and GRN→TNFRSF1A. Scores were computed on Gaussian spatial neighbor graphs (bandwidth 200 µm; cutoff 0.1) using the consensus L-R resource and bivariate (local cosine + global Moran’s I) statistics; permutation-based significance was assessed with 100 randomizations. Black stippling marks spots with permutation FDR < 0.05 (Moran’s I).

**Supplementary Figure 6. The impact of ARRDC3 overexpresses on the proliferation and migration of U251 cells.**


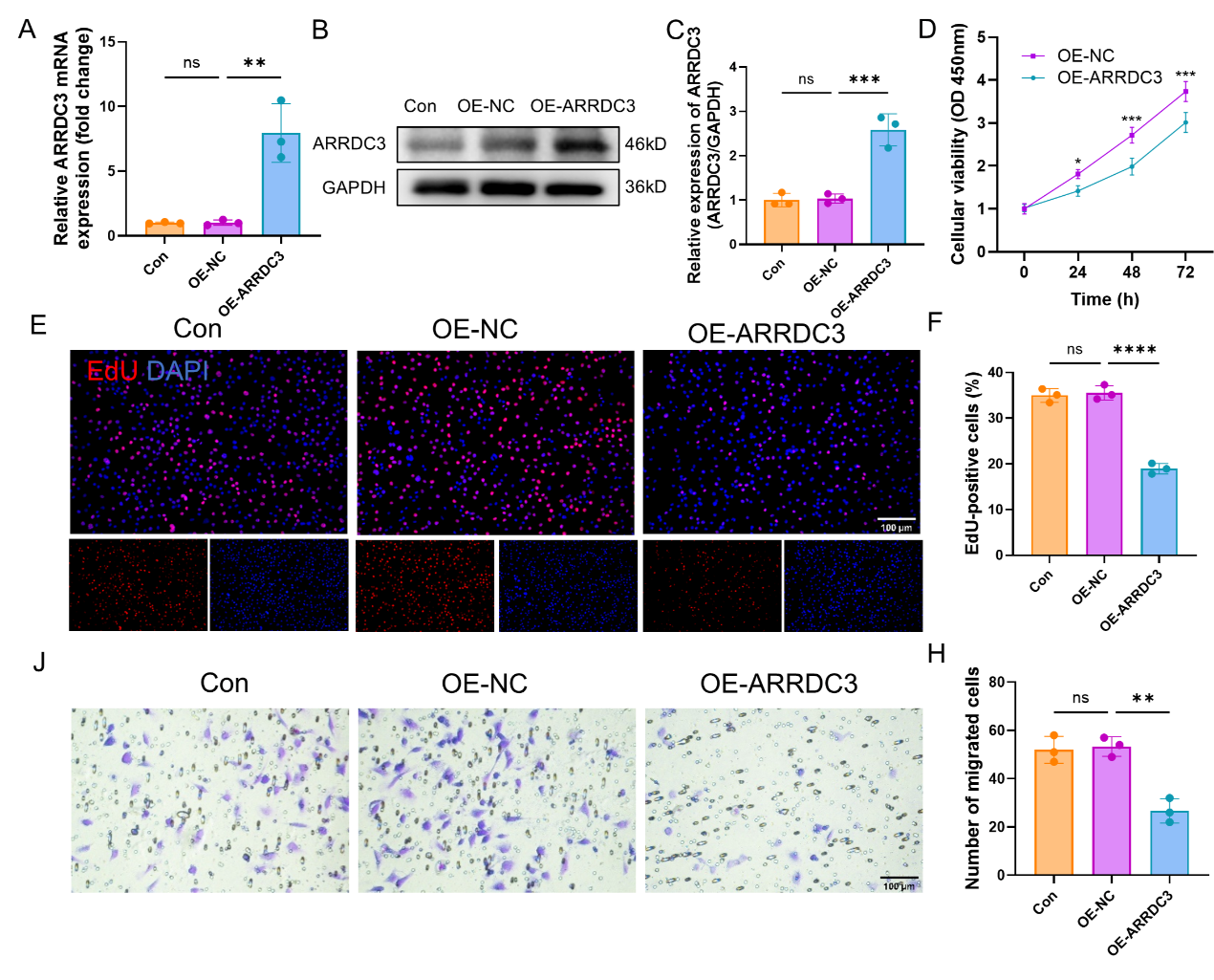


**Supplementary Figure 6. The impact of ARRDC3 overexpresses on the proliferation and migration of U251 cells.**

A, mRNA expression levels of ARRDC3 in U251 cells following transfection. B-C, Protein expression and quantitative results of ARRDC3 in U251 cells following transfection. D, CCK-8 reagent was added to the transfected U251 cells, and the corresponding absorbance values were measured at 24 h, 48 h, and 72 h at a wavelength of 450 nm. E-F, EdU incorporation assay assessing U251 cell proliferation, and quantitative analysis of EdU-positive cells relative to total DAPI-stained cells. G-H, The transwell migration assay was performed, and quantitative analysis was conducted to assess the number of migrated U251 cells.
