# Supplementary material for: Single-cell/spatial integration reveals an MES2-like glioblastoma program orchestrated by immune communication and regulatory networks
Source: Front Immunol. 2025 Oct 29;16:1699134. doi: 10.3389/fimmu.2025.1699134 (PMC12604987; doi:10.3389/fimmu.2025.1699134)
Supplement: Supplementary file 3 [file Table3.docx]

**Figure 11B**

**Original blot for ARRDC3**


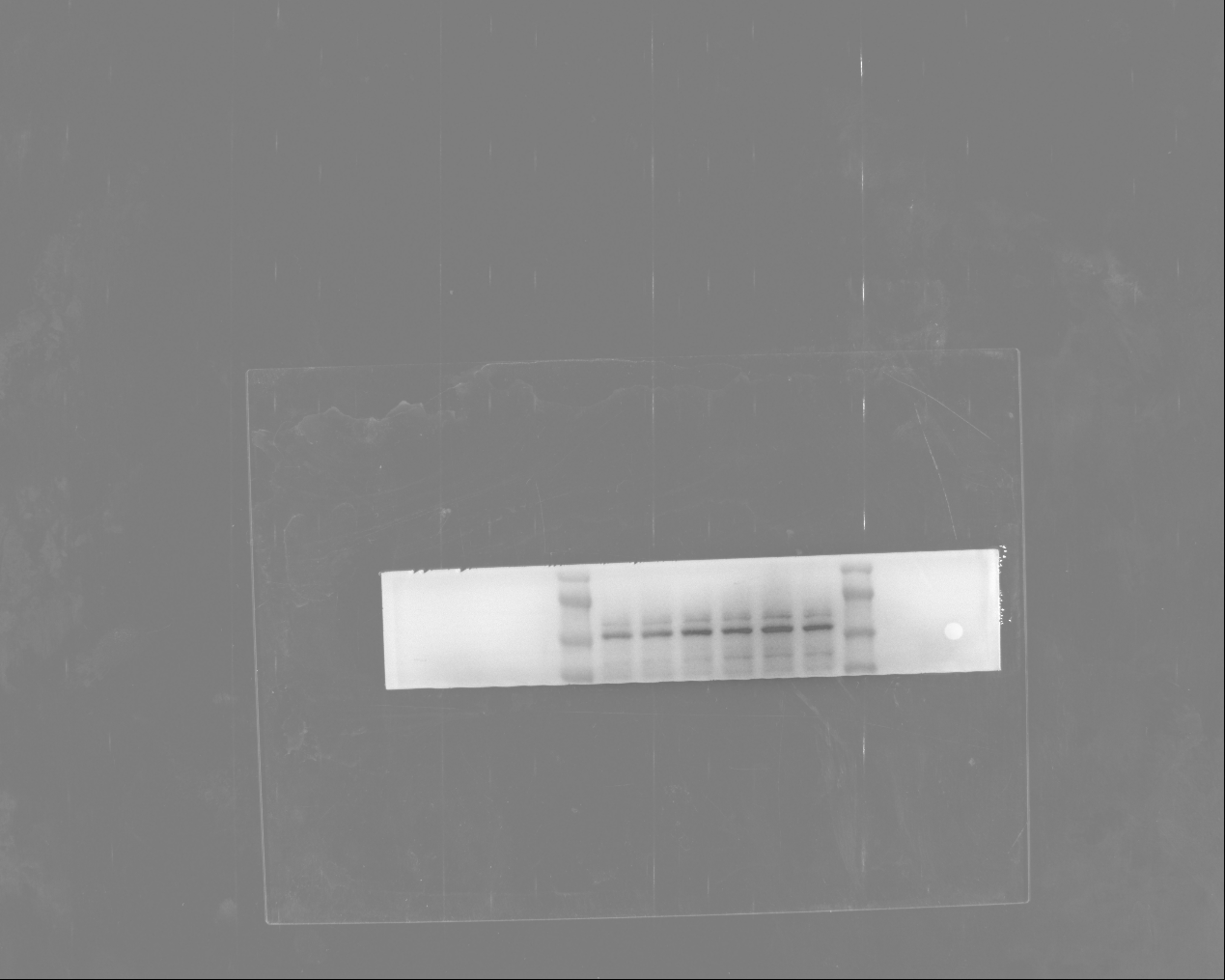


55 kD

43 kD

**Figure 11B**

**Original blot for GAPDH**

**
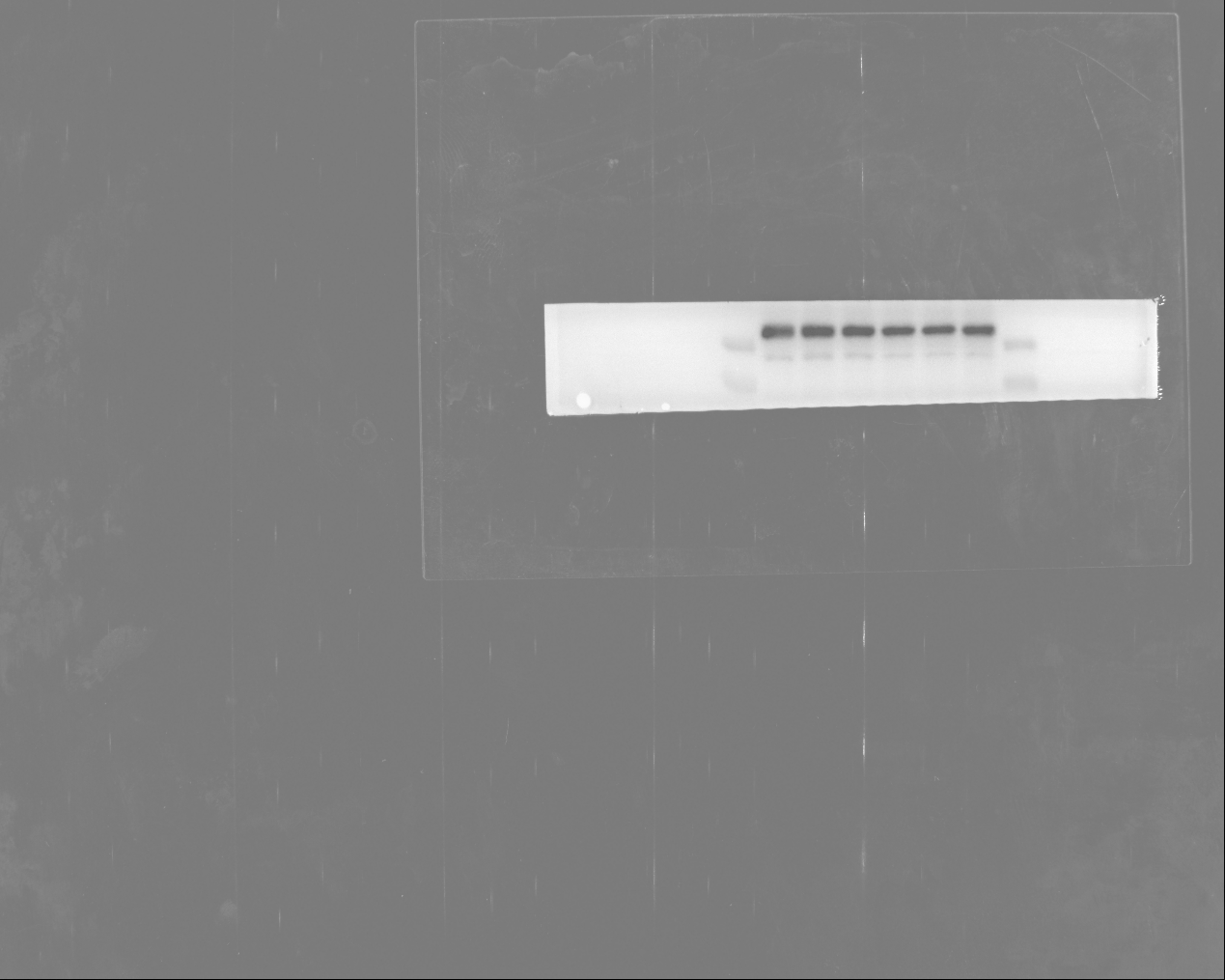
**

25 kD

33 kD

**Figure 11G**

**Original blot for ARRDC3**

**
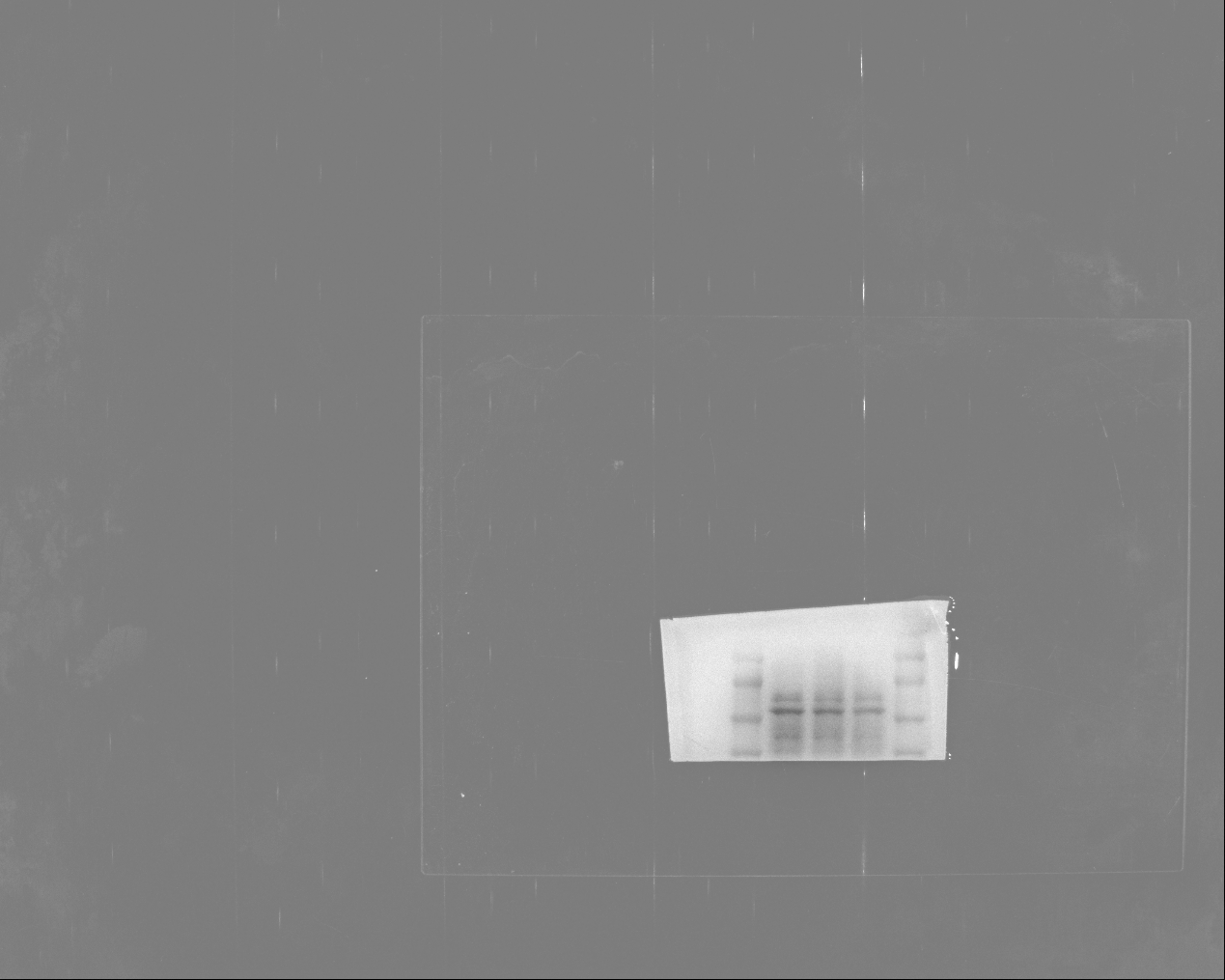
**

55 kD

43 kD

**Figure 11G**

**Original blot for GAPDH**


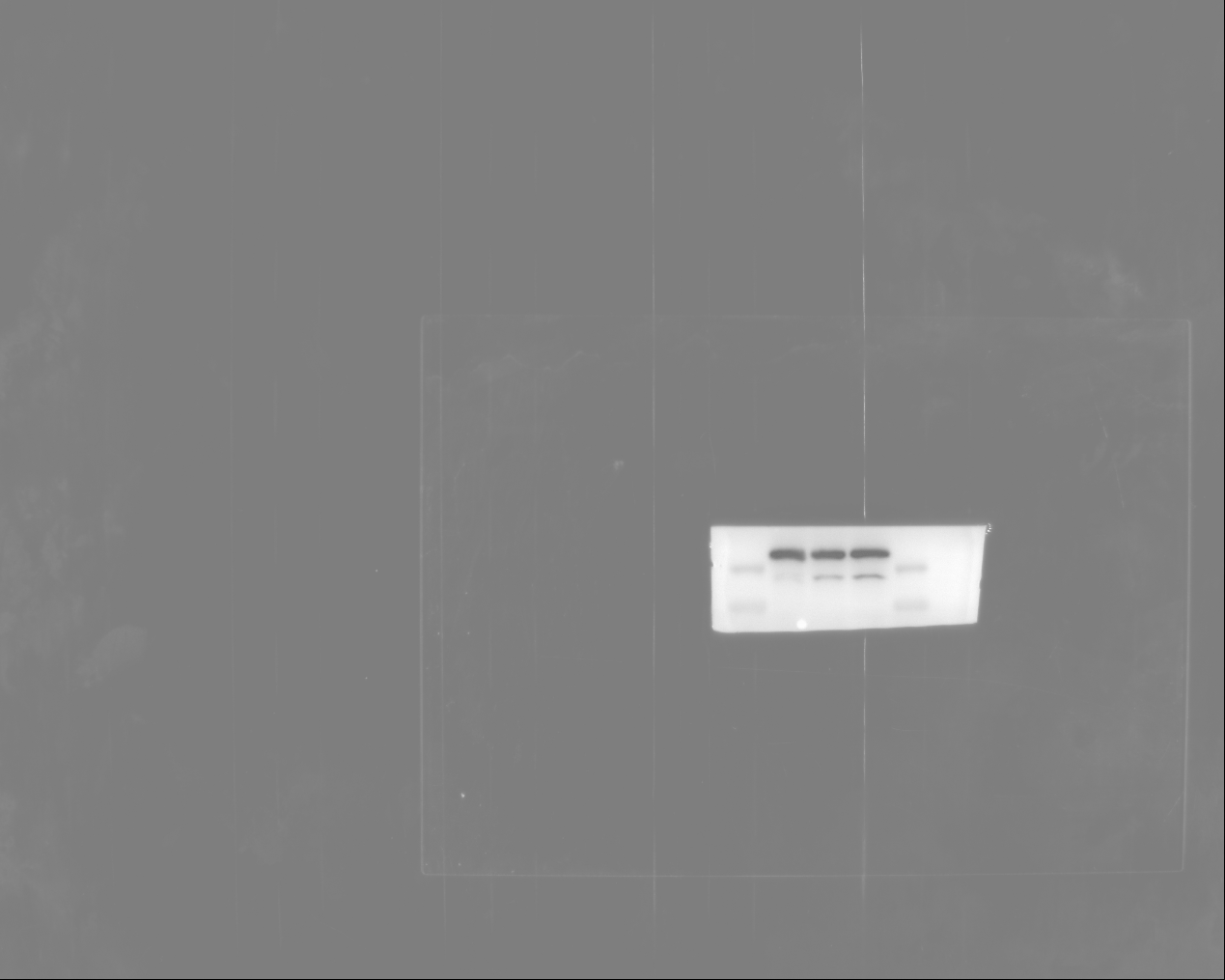


25 kD

33 kD

**Supplementary Figure 6B**

**Original blot for ARRDC3**


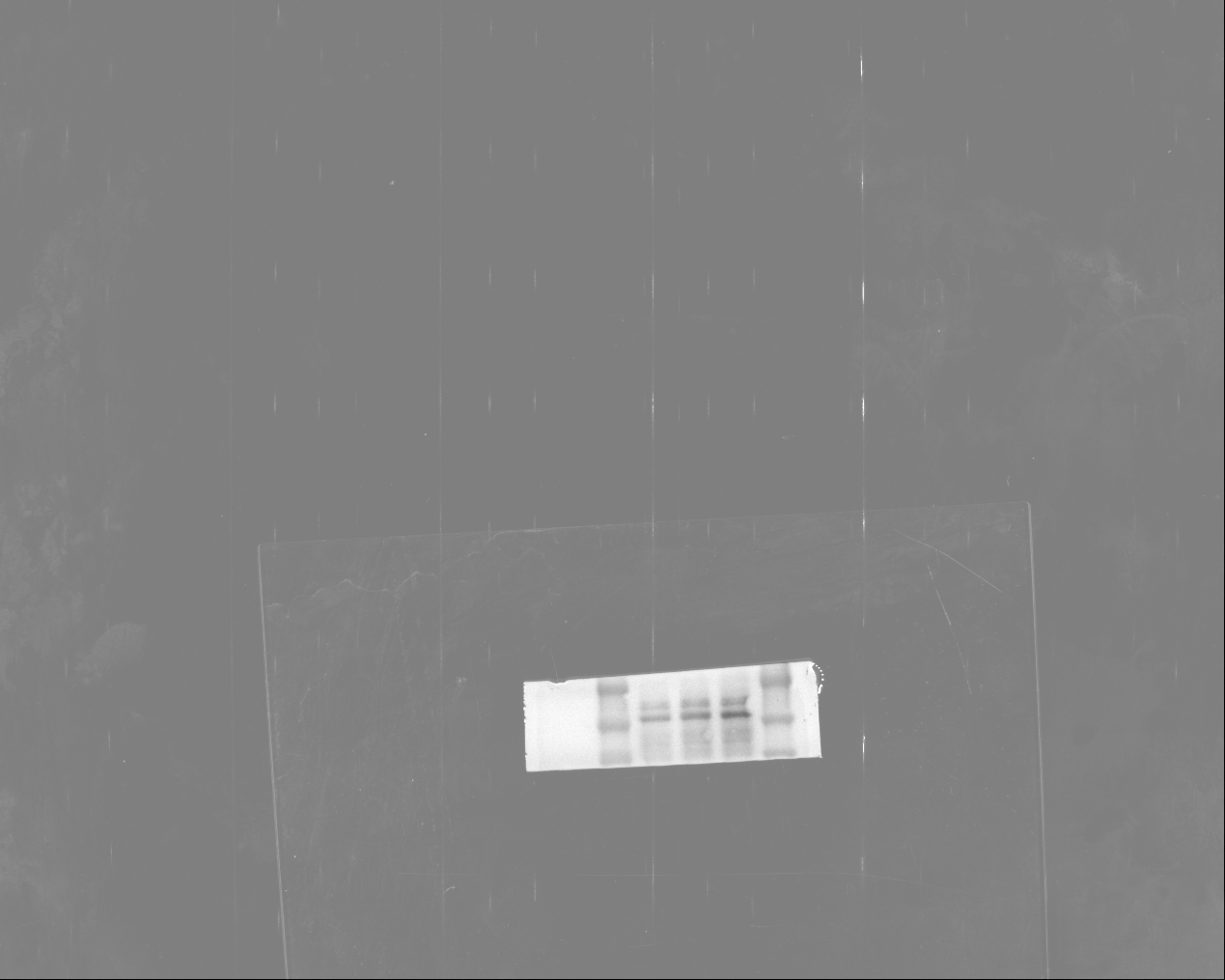


55 kD

43 kD

**Supplementary Figure 6B**

**Original blot for GAPDH**


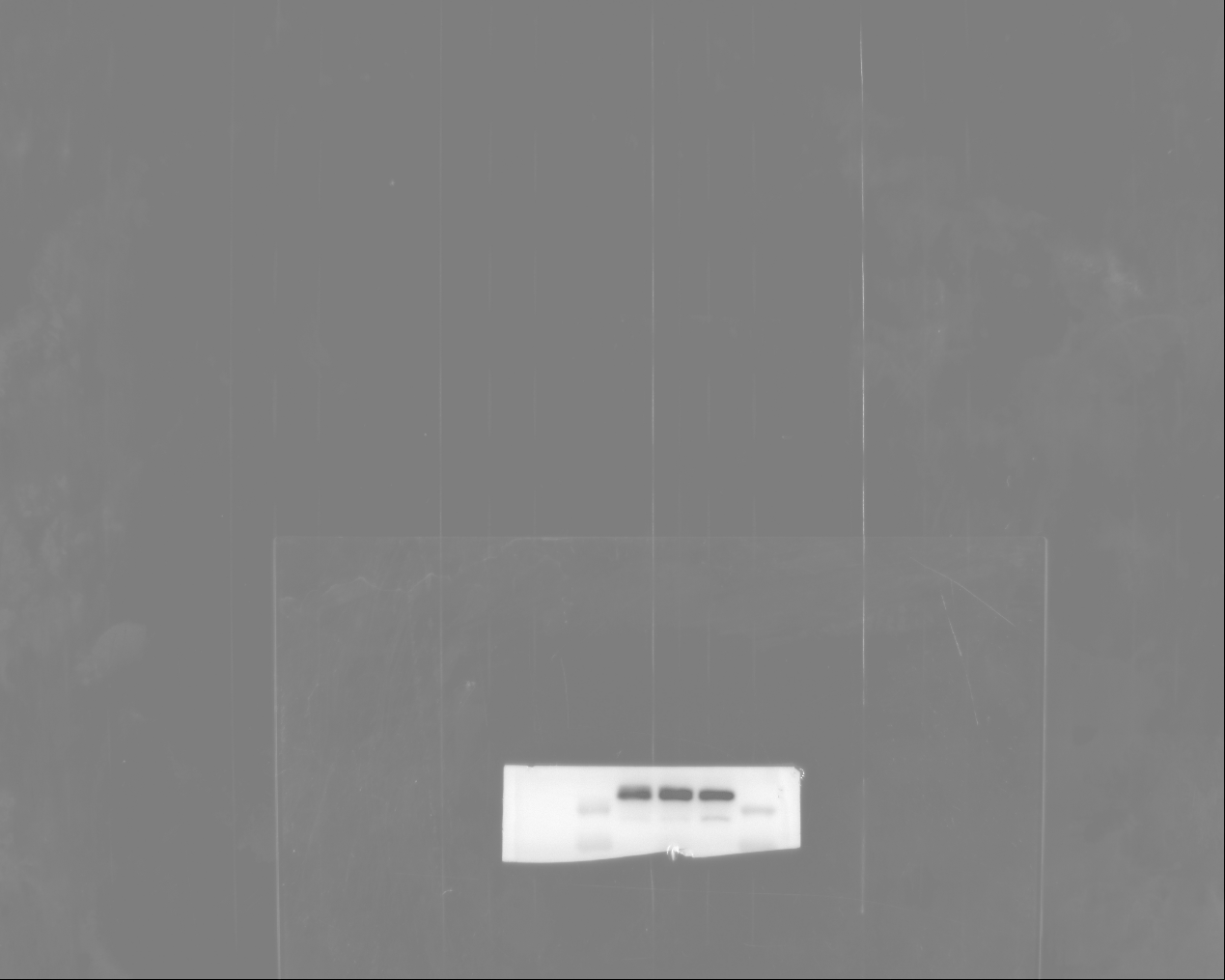


33 kD

25 kD
